# Phytotherapy and Nutritional Supplements on Breast Cancer

**DOI:** 10.1155/2017/7207983

**Published:** 2017-08-06

**Authors:** C. M. Lopes, A. Dourado, R. Oliveira

**Affiliations:** ^1^Fernando Pessoa Energy, Environment, and Health Research Unit/Biomedical Research Center (FP-ENAS/CEBIMED), Faculty of Health Sciences, Fernando Pessoa University, Porto, Portugal; ^2^EMAC (School of Alternative and Complementary Medicines), Porto, Portugal

## Abstract

Breast cancer is the most frequent type of nonskin malignancy among women worldwide. In general, conventional cancer treatment options (i.e., surgery, radiotherapy, chemotherapy, biological therapy, and hormone therapy) are not completely effective. Recurrence and other pathologic situations are still an issue in breast cancer patients due to side effects, toxicity of drugs in normal cells, and aggressive behaviour of the tumours. From this point of view, breast cancer therapy and adjuvant methods represent a promising and challenging field for researchers. In the last few years, the use of some types of complementary medicines by women with a history of breast cancer has significantly increased such as phytotherapeutic products and nutritional supplements. Despite this, the use of such approaches in oncologic processes may be problematic and patient's health risks can arise such as interference with the efficacy of standard cancer treatment. The present review gives an overview of the most usual phytotherapeutic products and nutritional supplements with application in breast cancer patients as adjuvant approach. Regardless of the contradictory results of scientific evidence, we demonstrated the need to perform additional investigation, mainly well-designed clinical trials in order to establish correlations and allow for further validated outcomes concerning the efficacy, safety, and clinical evidence-based recommendation of these products.

## 1. Introduction

Breast cancer is a significant public health problem in both developed and developing countries [1, 2]. Despite superior diagnostic skills and valuable advances in its treatment during the last decades, breast cancer persists in representing one of the most commonly diagnosed occurring cancers and leading cause of cancer deaths among women worldwide [3]. According to World Health Organization (WHO) it is estimated that worldwide over 508,000 women died in 2011 due to breast cancer [4]. The epidemiologic parameters (e.g., incidence, mortality, and survival rates) related to breast cancer diverge significantly between countries and regions [1, 5] which could be attributed to various factors such as health habits, lifestyle changes (e.g., dietary changes), exposure to radiation, family history, related alterations in menstrual cycle patterns, early detection, and access to the current knowledge concerning breast cancer [3, 5].

The stage of diagnosis influences both the prognostic and the treatment strategies for breast cancer. Currently, standard treatment protocol combines a multidisciplinary approach involving different therapies such as surgery, radiation, and medical oncology (i.e., chemotherapy, immunotherapy, and hormonal therapy) to obtain a local (i.e., remove or destroy cancer in the breast) or systemic (i.e., destroy or control cancer cells throughout the body) effect [3].

Despite the high incidence, breast cancer survivors, which used Complementary and Alternative Medicines (CAM), associated with standard cancer therapy, namely, chemotherapy and radiotherapy, are increasing [6, 7]. The use of CAM is growing among the public, up to 65% of the European population uses this modality of medicine, and it is commonly practiced among cancer patients [8]. Some studies associated the increased CAM use with sociodemographic issues such as female gender, higher levels of education, higher income, and health insurance [9–12] that explains its advance in many developed countries.

CAM is defined as a group of different modalities, including diverse medical and healthcare systems, products, and practices, which are not usually considered part of standard medical treatments [13]. This type of medicine could be used together with and thereby complement conventional medicine which is referred to as complementary medicine (e.g., using acupuncture to assist the side effects of conventional cancer treatment) or in place of conventional medicine (e.g., using a special diet to treat cancer instead of a conventional cancer treatment) [13, 14]. Despite alternative medicine being based on functional hypotheses often conflicting with conventional medicine, the complementary one uses the scientific approach of evidence-based medicine to support the conventional medicine. Currently an additional and promising term is emerging in this area, the “integrative medicine” which is based on the integration of conventional and complementary approaches together in a coordinated way that have been confirmed to be safe and effective [13, 15]. In CAM perspective, the patients are evaluated as a whole with all their complexities and connections instead of focusing on isolated pathological processes [15].

There are different classifications of CAM therapies which vary mainly with time and institutional approaches. In accordance with the National Centre for Complementary and Integrative Health, a reference USA Federal Agency, CAM therapies can be divided into three broad categories [13]:

- Natural products which include dietary supplements (e.g., vitamins, minerals, and probiotics) and phytotherapeutic products.
- Mind and body practices and manipulations which include different procedures or techniques such as yoga, chiropractic and osteopathic manipulation, meditation, massage therapy, acupuncture, relaxation techniques, tai chi, healing touch, qi gong, hypnotherapy, and movement therapies.
- Other complementary health approaches which include some approaches that may not neatly fit into either of the previous group, for example, traditional healers, Ayurvedic Medicine, Traditional Chinese Medicine, Homeopathy, and Naturopathy.

**Figure 1:**
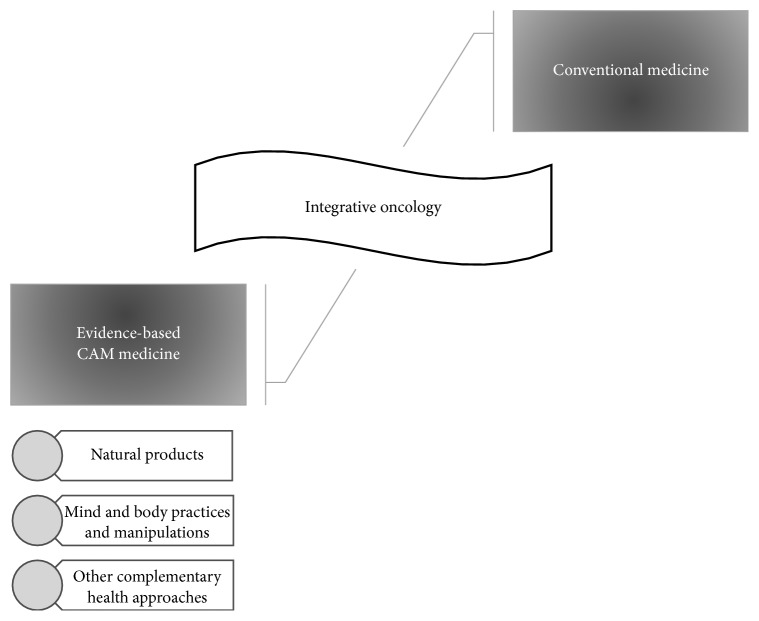
Components of integrative oncology (adapted from [16]).

In the oncology field, the patient survival rates have increased in recent years, so the practice of integrative care, termed integrative oncology [16] (Figure 1()), makes the acceptance of the holistic approach to cancer care by medical professionals feasible, once CAM modalities can meet various needs of the patients that go beyond the simple alleviation of severe side effects of conventional cancer treatments. This fact explains the use of CAM approaches by a great proportion of cancer patients [17, 18] and, among these patients, women with breast cancer remain the most likely users of some form of CAM modalities [12, 19–21] with an estimated rate as high as 75% [22]. Dobos et al. reported the practice of the concept of integrative oncology for breast cancer patients by German cancer centres such as the Department of Internal and Integrative Medicine, Kliniken Essen-Mitte, academic teaching hospital of the University of Duisburg-Essen, and the Breast Centre at Kliniken Essen-Mitte [16].

The reasons reported by breast cancer patients for the widespread use of CAM diverge and include [12, 19] activating the immune system, curing cancer, alleviating symptoms associated with the side effects of conventional cancer treatments, enhancing quality of life, increasing the perception of disease control, and preventing relapse and prolonging survival. Consequentially, the patients attempt to be active and gain autonomy [23].

However, the use of certain CAM methods in oncologic processes (i.e., a life-threatening disease) may become problematic and several partly substantial risks for the health of patient can arise, particularly when, as commonly happening, patients use them arbitrarily and do not report this information to their oncologists [19, 24–26]. This is true mainly for CAM recommendations or treatments that interfere/interact with chemotherapy or endocrine/hormonal treatment approaches, such as phytotherapeutic products and dietary supplements, or have intrinsic toxicity or other negative effects. Despite such interactions possibly being beneficial, in some situations, the concomitant use of CAM and conventional medicines approaches could compromise or be in conflict and enhance the drug toxicity or reduce the effectiveness [27]. A well-known example is phytoestrogens that might neutralize endocrine therapies. So, there are some CAM modalities that require a temporary adjustment of their use during periods of conventional treatment [28]. Additionally, among the CAM modalities, the consumer of these natural products is the most popular in breast cancer patients [19, 24] probably due to the assumption that “natural” products are less toxic than conventional prescribed medicines [29]. Therefore, attending the proactive role that nowadays the patients have in relation to their health, it is crucial to have reports in integrative medicine to guide and support clinicians and patients. The aim is to improve clinical/healthcare outcomes in combining CAM and conventional care and prevent misuse of CAM methods and preparations. The purpose is also to prevent harmful interactions and to enrich personal control over disease.

Based on the intensive investigation of nutritional supplements and phytochemicals as breast cancer therapeutics, the aim of this study is to compile and to explore the available scientific information regarding the most common phytotherapeutic and nutritional supplement products used in breast cancer patients. Therefore, recent scientific evidence studies (e.g., systemic reviews and clinical investigation studies) are consulted and the clinical relevant and validated outcomes concerning efficacy, safety, and limitations of the clinical data are reported.

## 2. Methodology

To elaborate this review, PubMed (indexed for MEDLINE) and ISI Web of Science were searched using the following key words: breast cancer; phytotherapy; supplements; CAM; integrative medicine;* Echinacea*;* Tabebuia impetiginosa; Salvia; Uncaria; Allium sativum *L*., Linum usitatissimum; Curcuma; Camellia sinensis; Ginseng; Cimicifuga racemosa; Viscum album; *vitamins; antioxidants; vitamin A; *β*-carotene; vitamin C; vitamin E; vitamin D; selenium, calcium; B complex; omega 3. For plants, both Latin designations and common trivial names were considered for search strategy. Additionally, text books were explored and reference lists from pertinent reviews were scrutinized. The literature search was confined to the period between 2000 to March of 2017. Systematic reviews, meta-analyses, and* in vivo* and relevant cell line studies were considered for this review.

## 3. Phytotherapy

Among CAM used in cancer patients, herbal preparations or phytotherapy is the most commonly and the oldest used group of treatment [30]. Most of time, patients use plant products for self-medication. It uses products derived from all or parts of plants and is a common practice in all civilizations around the world including Asia, Africa, Europe, and America. Herbal preparations may have superior risk of adverse effects and therapy interactions than other complementary therapies due to the potential active ingredients of various plants. Despite this, phytotherapeutic products are not tested with the scientific rigor required of conventional drugs nor are controlled by any purity and potency certificate [3].

The recognition of medicinal plants as effective and inexpensive sources of synthetic novel chemotherapeutic compounds is increasing in the last decades and many researchers focus their research on this promising area [31]. In the cancer domain, the biological effects of herbal medicinal products could be diverse such as [7] defence from malignancy by increasing detoxification or cleaning, modification of the action of some hormones and enzymes, reduction in side effects and complications of chemotherapy and radiotherapy, and improvement of the function of the body's immune cells (i.e., stimulates the production of cytokines including interleukin, interferon, tumour necrosis factor, and colony stimulating factor).

The reasons for using phytotherapeutic products include [3] to lessen symptoms of disease and to prevent disease (e.g., garlic contains high levels of organosulfur compounds that have been experimentally proven to prevent cancer in animals [32]).

In a prospective study using an exploratory analysis, the authors found that some evidence that phytotherapeutic products use among long-term breast cancer survivors (for at least 10 years) was associated with inferior survival rates and a poorer physical component score [30]. The most frequent phytotherapeutic products used among long-term (at least 10 years) breast cancer survivors who participated in this study were* Echinacea*, herbal teas, and ginkgo biloba. Authors reported limitations in the study such as few deaths for mortality analysis and lack of information on when phytotherapeutics use was initiated, duration, or application. In another study, McLay et al. [33] reported that 38% of treated breast cancer patients (in a total of 360 questionnaires) use herbal preparations (*Echinacea*, pomegranate, peppermint, chamomile, grapefruit, garlic, and ginseng) that have the potential to interact with adjuvant endocrine therapies (e.g., tamoxifen, anastrozole, letrozole, and exemestane). Garlic, gingko, and* Echinacea* were the most frequently phytotherapeutic products among African Americans (Black Women's Health Study) [34].

### 3.1. *Echinacea*

*Echinacea*, a member of the family* Asteraceae*, has a long history of medicinal use. It is endemic to eastern and central North America and is also cultivated in Europe. Three different species of* Echinacea* can be used as phytotherapeutic products:* Echinacea purpurea, Echinacea angustifolia*, and* Echinacea pallida* [3].

Some authors justified the potential use of* Echinacea* as an anticancer agent based on its rich content in flavonoids that act as an immune-stimulant by promoting the activity of lymphocytes thus increasing phagocytosis and the activity of natural killer cells and inducing interferon production [35].

Although studies indicated the use of* Echinacea* among breast cancer patients [30, 33, 34], there are not many studies, even* in vitro*, that demonstrated its effects in this type of cancer. Driggins et al. verified that despite* Echinacea pallida* decreasing the growth rate of BT-549 mammalian breast cancer cells, its effect was significantly lower as compared to* Echinacea purpurea *[36]. Huntimer and collaborators used* Echinacea angustifolia* roots and evaluated their activity when combined with doxorubicin (i.e., cytotoxic agent) in MCF-7 human breast cancer cell line [37]. This study showed that different constituents of* Echinacea* could have a different effect on MCF-7 cell proliferation and could interfere with cells treated with anticancer drug, affecting cell proliferation despite the presence of doxorubicin (i.e., counteracting the cell-killing activity of doxorubicin). Based on this effect, the authors suggest that herbal medicines need to be examined more closely for interactions with other chemotherapeutic agents.* Echinacea* induce cytochrome P450 3A4 isoenzyme system both* in vitro* and in humans [38, 39]. This enzyme system participates in the metabolism of many chemotherapeutic agents. Goey et al. demonstrated that the recommended dose and schedule of a commercially available* Echinacea purpurea *extract (A. Vogel Echinaforce®, Biohorma BV, Elburg, Netherlands) did not interact with docetaxel pharmacokinetics and this combination can be used safely [40]. Among other therapeutic indications, docetaxel is approved for the treatment of locally advanced or metastatic breast cancer. The benefits of the use of* Echinacea *to reduce unwanted effects of radiotherapy (e.g., leukopenia) are unclear [41]. Therefore, more clinical evidence is important to support or refute the recommendations for* Echinacea* in relation to cancer management.

Even though* Echinacea *seems to be relatively safe, it may cause liver damage or suppress the immune system if used for a prolonged period without a break (i.e., more than 8 weeks) [42]. Therefore, a patient with liver disturbance or taking drugs that potentially cause liver toxicity (e.g., some chemotherapy agents) should avoid* Echinacea* use.

### 3.2. Lapacho

Lapacho tree or pau d'arco is the common name of* Tabebuia impetiginosa* Martius ex DC species, the family of Bignoniaceae. It is a tree indigenous to the Amazonian rainforest and other regions of South America and Latin America. Pau d'arco has been used in traditional medicine for many centuries due to its different physiological effects such as fungicide, antibacterial, antiviral, anti-inflammation, and anticancer [43]. Above all, special attention has been given to the antitumour activity of *β*-lapachone (i.e., a constituent of lapacho) against many* in vitro* cancer cell lines, including breast cancer [44, 45] due to its action on reinforcing the immune system.

The clinical evidence of the health benefits of lapacho is restricted to studies related to its potential anticancer effects in phase I and II clinical trials [46]. However, this effect was not borne out by clinical trials [43]. The Food and Drug Administration (FDA) registered it as a dietary supplement with the following recommendation “to alleviate conditions and symptoms of cancer.”

Despite the underlying mechanism being under investigation [47], the cytotoxic effects to some cancer cells, including breast cancer cell lines, are confirmed [45, 48, 49]. *β*-Lapachone also sensitizes the response of different cancer cell lines to ionizing radiation [50, 51], interacting synergistically with this conventional cancer therapy. Bey and collaborators showed that the combination of *β*-lapachone and radiation exert synergistic effects against human mammary epithelial cells (HMEC 1585), in which *β*-lapachone sensitizes cells to radiation by inhibiting DNA repair, and radiation sensitizes cells to *β*-lapachone by increasing oxidoreductase enzyme, which reduces *β*-lapachone to an unstable semiquinone level, in tumour cells [51].

Concerning the toxicity issues of lapacho, limited data are available and more clinical trials are required to evaluate the toxicity of *β*-lapachone toward normal human tissue and to establish the best dosage range [52].* Tabebuia impetiginosa* tea emerges as generally safe and has a FDA regulatory classification of “generally recognized as safe” (GRAS) status. Recently, Lemos et al. [53] demonstrated genotoxic effects in rats at a comparatively high dose range. The most important interaction of this botanical product refers to the interference in the biological cycle of vitamin K [46]. It is also important to attend the variable quality and composition of the herbal products commercially available.

### 3.3. *Salvia*

*Salvia* is the largest and the most important genus of the family Labiatae [89]. This genus includes wild growing and cultivated medicinally valuable species (e.g.,* Salvia bracteata* and* Salvia rubifolia*) as well as ornamentals.* Salvia *species present a high diversity in their secondary metabolites (e.g., flavonoids, diterpenoids, volatile oils, and tannins) which justify the multiple pharmacological effects reported in the literature [90].

In breast cancer, different species were investigated for their* in vitro* antiproliferative activity. Abu-Dahab et al. [90] demonstrated that the ethanol extract of three species, namely,* S. syriaca*,* S. fruticosa, *and* S. horminum*, presented selective antiproliferative activity against oestrogen receptor (ER) positive breast cancer cell lines with minimum toxicity against normal human periodontal fibroblasts. Based on their safe and selective effects, the authors suggested the use of these* Salvia *species as promising plant-originated anticancer agents. Other species also showed promising results.* S. triloba* and* S. dominica* ethanol extracts showed antiproliferative effects on adenocarcinoma of breast cell line (MCF7, oestrogen receptor-positive) and human ductal breast epithelial tumour cell line (T47D) via proapoptotic cytotoxic mechanisms [91].* S. miltiorrhiza* (i.e., Danshen which is widely used in traditional Chinese medicine) exhibited a strong inhibitory effect on the proliferation of MCF-7 breast cell line and induced cell cycle delay in the G1 phase via modulation of Akt phosphorylation and p27 level [92]. Authors also used MCF-7 HER2 cell line which over expresses HER2. HER2 (i.e., human epidermal growth factor receptor type 2) is a receptor tyrosine kinase and is involved in signal transduction pathways leading to tumour cell proliferation. HER2 is overexpressed in a high percentage of breast cancer (25–30%) and its overexpression is associated with aggressive tumours, a high rate of metastasis and relapse, poor prognosis, and limitation in treatment (in most cases it became resistant to endocrine therapy such as tamoxifen) [93, 94]. The MCF-7 HER2 cells were more resistant to the Danshen actions.

Danshen extracts contain diterpene quinone and phenolic acid derivatives such as tanshinone (I, IIA, and IIB), cryptotanshinone, isocryptotanshinone, miltirone, tanshinol (I and II), and salviol [95]. These compounds are antioxidant agents and protect against lipid peroxidation. Some of these compounds have been isolated from Danshen, sometimes synthesized, and their* in vitro* cytotoxic activity tested against diverse cancer cell lines, including breast cancer [96–99]. Besides the* in vitro* inhibition of ER-positive human cancer cells lines, Wang and collaborators also proved that neotanshinlactone was more potent and more selective than tamoxifen citrate [96]. In this area, only one* in vivo* study has reported anticancer activity on mice bearing human breast infiltrating duct carcinoma orthotopically [95], where the compound tanshinone II A strongly inhibited the* in vitro *proliferation of ER-positive breast cancer cells and inhibited* in vivo* growth of ER-negative breast cancer. The inhibition of proliferation and apoptosis induction of cancer cells through upregulation and downregulation of multiple genes involved in cell cycle regulation, cell proliferation, apoptosis, signal transduction, transcriptional regulation, angiogenesis, invasive potential, and metastatic potential of cancer cells could explain in part the anticancer effect of this compound. Chemotherapy resistance is a significant problem in breast cancer therapy. Cai et al. reported the reversal mechanism of salvianolic acid A (i.e., a phenolic active compound extract from* Salvia miltiorrhiza*) in human breast cancer paclitaxel resistance cell line, facilitating the sensitivity of chemotherapeutic agents [100]. In another study, the authors demonstrated that tanshinone II A ameliorated hypoxia-induced chemotherapy resistance to doxorubicin and epithelial-mesenchymal transition in breast cancer cell lines via downregulation of hypoxia-induced factor 1*α* expression [101]. However,* in vivo* studies are required to support these achievements.

Wong et al. performed a clinical trial and concluded that the coadministration of* Coriolus versicolor* (Yunzhi, 50 mg/kg body weight, 100% polysaccharopeptide) and* Salvia miltiorrhiza* (Danshen, 20 mg/Kg body weight) could be a promising approach to improve immunological function in posttreatment breast cancer patients [54]. Patients supplemented for 6 months presented significantly elevated values of absolute counts of T-helper lymphocytes, the ratio of T-helper/T suppressor and cytotoxic lymphocytes, and the percentage and absolute counts of B-lymphocytes and decreased values of plasma sIL-2R concentration. In other clinical study, the intravenous administration of* Salvia miltiorrhiza* extract was able to reduce ischemia and necrosis of skin flaps after mastectomy as well as anisodamine administration but with no adverse effects [55].

### 3.4. *Uncaria*

Two species of* Uncaria*, commonly known as cat's claw,* Uncaria guianensis *and* Uncaria tomentosa*, found in northern regions of South America and belonging to Rubiaceae family, have also promising medicinal outcomes, including in breast cancer patients, due to their immune-stimulant and antioxidant properties [102]. This botanical product contains a complex combination of phytochemicals, including glycosides, tannins, flavonoids, and sterol fractions that could be complementary and/or synergic in their pharmacological actions [3]. Some of these constituents can present selectively cytostatic/cytotoxic to some cancer cells such as pentacyclic oxindole alkaloids [103].

Although some studies revealed the* in vitro* efficacy of cat's claw in breast cell lines [102, 249] no clinical trials investigating* Uncaria* species as an anticancer agent are available. It is fundamental that more research is performed in animal models and mainly in humans before any conclusions can be drawn in this topic.

Utilising* Uncaria tomentosa *appears to be a beneficial approach to minimize the adverse effects associated with traditional cancer therapies, namely, in the case of chemotherapy. The use of this* Uncaria* species can stimulate DNA restoration [250], preventing mutations and cell damage caused by chemotherapy agents [251], and myelopoiesis [252, 253]. Aqueous extracts of* U. tomentosa *also proved to improve leukocyte counts during a period of eight weeks in healthy animals [254] and after ten days of doxorubicin-induced neutropenia [251]. In addition, extracts or fractions of cat's claw modulate the activity of the immune system [254, 255]. These preclinical data were proved in a randomized clinical trial. Santos Araujo Mdo et al. used 300 mg per day of* U. tomentosa *dried ethanolic extract, in patients diagnosed with Invasive Ductal Carcinoma Stage II, who underwent a treatment regimen known as FAC (Fluorouracil, Doxorubicin, and Cyclophosphamide) [56]. This adjuvant treatment for breast cancer patients was safe and effective in the recovery from neutropenia induced by cancer chemotherapy. The dose used in this trial was empiric and was based on the dose administrated in other (not cancer-related) clinical trials where the authors used different solvent extracts and, consequently, different phytochemicals. So, more clinical trials should identify the best dosage range for using cat's claw as an adjuvant chemotherapy agent.

Budán et al. indicated that the combination of different phytotherapeutic (e.g., Clae of Dragon tea containing the bark of* Uncaria guianensis*,* Uncaria tomentosa*, and* Tabebuia avellanedae*) in a long-term experimental animal model acted as chemopreventive agent [256].

Concerning their safety, clinical trials with human volunteers reported no toxicity associated with the use of a commercially available aqueous extract of* U. tomentosa *named C-Med-100. The dose of* Uncaria* was different in the trials, using 250 mg or 350 mg/day over 8 weeks and 2 × 350 mg daily for 2 months [250, 257].

Cat's claw could provoke adverse effects including diarrhoea or loose stools and lower blood pressure, which tend to diminish with continued usage. However, some literature reported that cat's claw can interact with medications intended to suppress the immune system (e.g., cyclosporine) or other medications prescribed following an organ transplant; this information still needs to be proven scientifically.* In vivo* rat studies demonstrated that cat's claw may protect against gastrointestinal injury attributed to nonsteroidal anti-inflammatory drugs (NSAIDs) and can diminish the platelet aggregation and may increase the effect of anticoagulants [103].

### 3.5. *Allium sativum* L

*Allium sativum*, commonly known as garlic, presents different biologically useful secondary metabolites with high sulphur content, such as S-allyl-cysteine, diallyl disulphide, diallyl trisulfide, and methyl allyl trisulfide [258]. Garlic also contains other beneficial compounds such as arginine, oligosaccharides, flavonoids, and selenium (i.e., cellular antioxidants) [259]. The main active ingredients of garlic, organic sulphur compounds, have attracted great attention as cancer prevention and treatment agents in breast cancer [260–263]. Among these constituents derived from garlic, the oil-soluble compounds are more effective than water-soluble compounds in suppressing breast cancer [264]. The mechanisms involved in the anticancer effect of garlic-containing compounds include activation of metabolizing enzymes that detoxify chemical carcinogens, inhibition of DNA adduct formation, suppression of reactive oxygen species production, induction of apoptosis, and regulation of cell cycle progression and signal transduction modification [264]. All referred to studies used experimental breast cancer cell lines, other studies extended their anticancer evidences to* in vivo* models [265, 266], and no clinical trials are available in literature. For example, Liu et al. [260] demonstrated that diallyl trisulfide, a natural organosulphuric compound with most sulphur atoms found in garlic, suppressed the migration and invasion of breast cancer cell lines (MDA-MB-231 cells and HS 578t cells) and suggested that the inhibitory effects are associated with downregulation of the transcriptional activities of nuclear factor-kappa (NF-*κ*B, a transcription factor that regulates the expression of antiapoptotic proteins) and ERK/MAPK (i.e., major kinases involved in cell survival) signalling pathways. In many malignant tumours, constitutive NF-activation occurs and consequently inflammation, proliferation, resistant to apoptosis, invasion, and so forth [267]. These authors reported that a concentration of diallyl trisulfide equal to 10 *µ*M should be achieved* in vivo* for preventing or treating breast cancer. Chandra-Kuntal and collaborators established the critical role for reactive oxygen species in the anticancer effects of diallyl trisulfide compound using human breast cancer cells (MCF-7 and MDA-MB-231). Using an oestrogen receptor-negative human breast cancer cell line (MDA-MB-231), Nakagawa et al. [266] reported that diallyl disulphide synergizes the effect of eicosapentaenoic acid, a breast cancer suppressor, and antagonizes the effect of linoleic acid, a potent breast cancer stimulator. Diallyl trisulfide inhibits the expression of ADAM10 and ADAM17 (proteases with a role on metabolism of abnormal cells and whose high expression is associated with a lower disease-free survival in breast cancer patients) in estrogen-independent MDA-MB-231 and estrogen-dependent MCF-7 breast cancer cells and seems to promote growth inhibition of breast cancer cells [268].

In terms of the cancer prevention, Wargovich et al. demonstrated robust chemopreventive action of constituents of garlic against experimentally induced cancer, including the mammary gland [32].

Despite garlic affecting cytochrome P450 3A4 activity, Cox et al. showed that garlic supplementation does not significantly affect the disposition of docetaxel but it can decrease the clearance of docetaxel in patients carrying a* CYP3A5∗1A* allele (present in all African American) [58].

A case-control study performed in northwest Iran aimed to find the association between dietary* Allium* consumption and risk of breast cancer. The study included 285 women (25–65 years old) diagnosed with histopathologically confirmed breast cancer (grade II or III or clinical stage II or III) which completed a food-frequency validated questionnaire. A reduced risk of breast cancer associated with higher consumption of garlic and leek and an increased risk associated with high consumption of cooked onion was found [57].

No interactions are reported. Theoretically, garlic can increase bleeding with anticoagulants, aspirin, and antiplatelet drugs [269].

### 3.6. *Linum usitatissimum*

*Linum usitatissimum* (flaxseed) is known for its phytoestrogen lignans content, namely, secoisolariciresinol diglucoside, which are converted into mammalian lignans (enterolactone and enterodiol) by bacterial fermentation in the colon [270]. This bacterial conversion beneficially influences the anticancer effects of flaxseed [271]. Based on their structural similarity to estrogens, mammalian lignan metabolites can attach to oestrogen receptors and inhibit the growth of estrogen-stimulated breast cancer [3]. Flaxseed can modulate the estrogen metabolism and oestrogen receptor and epidermal growth factor receptor signalling pathways [272]. Flaxseed also contains up to 40% oil which is mainly rich in *α*-linolenic acid-rich oil (i.e., *n*-3 polyunsaturated fatty acid).

However, some questions remain and are discussed, such as if flaxseed and its compounds are effective in reducing the breast cancer risk, present antiproliferative properties, and can interact beneficially with conventional cancer therapy.

In 2013, a Canadian study revealed that flaxseed intake alone is associated with a prevention of breast cancer [59].

*In vitro* studies showed that flaxseed induces apoptosis and inhibits human breast cancer cells proliferation [273–276]. Animal models have shown that flaxseed, secoisolariciresinol diglucoside, and flaxseed oil can reduce the growth of breast cancer [277–279]. Additionally, experimental studies using rodents demonstrated that flaxseed dietary inclusion has antiproliferative effect in different heterotransplanted mammary carcinomas in mice [280–282]. For example, Chen et al. proved that flaxseed diet on a mouse model has a dose-dependent inhibition of breast tumour growth [283]. In addition, flaxseed also contributed to decreased metastasis and tumour angiogenesis [60, 279, 284].

Even though numerous experimental studies using animal models being available in literature, there are a few studies concerning the influence of flaxseed on breast carcinomas in humans and more clinical trials are required to assess whether flaxseed has anticancer properties in humans. No study reveals that flaxseed has a negative effect. For example, in a double-blinded, randomized controlled clinical trial, the dietary flaxseed demonstrated remarkable protection with a reduction in tumour growth and alteration of tumour biological markers in postmenopausal breast cancer patients [285]. Buck and collaborators [63, 286] also reported the beneficial effect of flaxseed ingestion and high serum lignan levels in the survival rate of postmenopausal patients with breast cancer.

Taking into consideration the interaction of flaxseed in chemotherapy, Chen et al. demonstrated that *n*-3 fatty acid-rich cotyledon fraction of flaxseed reduced the growth of ER-positive human breast tumours, alone and in combination with tamoxifen, increasing the effectiveness of this chemotherapeutic agent [280]. Some studies reported the decreased tumour angiogenesis with the association of flaxseed and tamoxifen [60, 61] and the tumour cell apoptosis with flaxseed and doxorubicin [287]. In a recent study, Manson et al. reported that dietary flaxseed presented minimal tumour-reducing outcome did not interfere with trastuzumab action (a recombinant humanized monoclonal antibody used as the first-line therapy in HER2-overexpression breast cancer) but enhanced survival in athymic mice with established HER2-overexpressing human breast tumours [288]. However, the use of flaxseed oil combined with trastuzumab increased the effectiveness of low doses of this monoclonal antibody, that is, reduced tumour size and cell proliferation and increased apoptosis on HER2-overexpressing breast tumours (BT-474) in athymic mice compared to trastuzumab alone [289]. The author suggests the potential use of flaxseed oil as a complementary treatment for premenopausal women undergoing trastuzumab treatment, reducing the dose, and, therefore, lowering the side effects and potentially increasing survival rates. However, these recommendations should be confirmed through clinical trials. The use of flaxseed and aromatase inhibitor (using anastrozole as model drug) was also studied by MaCann and collaborators using biopsy and resection samples from postmenopausal women with oestrogen receptor-positive breast cancer [62]. Nevertheless, the results did not support strong effects on aromatase inhibitor activity but suggested that anastrozole might reduce the beneficial effects of flaxseed.

Additionally, Chen et al. verified that flaxseed components (secoisolariciresinol diglucoside and oil) did not attenuate the positive effects on bone health induced by tamoxifen (i.e., increase bone mineral content and density) in breast cancer patients [290].

Based on the current evidence, the flaxseed and its components are safe and effective in reduction risk and treatment of breast cancer. Despite this, the use of flaxseed is associated with bowel obstruction and bleeding disorder [3].

### 3.7. *Curcuma longa*

Turmeric plant* (Curcuma longa)* is widely used in food as a dietary spice and in traditional medicine as a remedy for different diseases including diabetes and hepatic disturbances [291]. Curcumin, the active compound of turmeric, has antioxidant effects and has been demonstrated to be a promising agent in clinical oncology due to its chemopreventive, antiproliferative, and apoptosis effects [292].

Curcumin can modulate multiple biological pathways involved in mutagenesis, oncogene expression, cell cycle regulation, apoptosis, angiogenesis, tumour genesis, and metastasis, which could justify its anticancer outcome [293].

Various preclinical studies focused on the anticancer efficacy of curcumin have been tested in some cancer models including breast cancer. The inhibition effect of curcumin alone, in combination with chemotherapeutic agents, on* in vitro* human breast cancer cells lines has been proved [294–299]. The same effects have also been observed in animal models. Zhan and collaborators [296] demonstrated the increased antitumour efficacy on mouse models of combining paclitaxel with curcumin and suggested the promising therapeutic potential and underlying mechanisms of this therapeutic association in breast cancer treatment. Ferreira et al. reported the effectiveness in reducing tumour growth and cell proliferation as well as in the suppression of angiogenesis using intraperitoneally curcumin administration in a xenograft model of breast cancer [300]. Research studies referred to different molecular mechanisms underlying the antitumour activity of curcumin in breast cancer cells, such as modulating the NF-*ƙ*B signalling pathway [296, 297, 301–303], decreasing HER2 oncoprotein expression, the phosphorylation of Akt, MAPK, the expression of NF-*ƙ*B in both BT-474 and SK-BR-3-HR cell (i.e., a herceptin resistant strain from SK-BR-3) [304], and the induced apoptosis by inhibiting fatty acid synthase [305]. Additionally, some authors found that curcumin suppressed breast tumour angiogenesis through abrogating osteopontin or medroxyprogesterone acetate induced VEGF expression [306, 307]. Soung and Chung showed that the association of epigallocatechin gallate and curcumin has an efficacy outcome in both* in vitro* and* in vivo* models of ER*α*-breast cancer by the regulation of epidermal growth factor receptor expression [308].

Curcumin is a lipid-soluble compound with limited bioavailability and extensive metabolization. Some researchers used different technological strategies to sustain the delivery of curcumin and overcome the intrinsically poor bioavailability, such as nanotechnology and liposomal-based formulations and synthetic analogues of curcumin [309–313]. The results from these studies demonstrated promising outcomes for clinical transposition.

Most of the clinical trials that evaluated the curcumin used in cancer treatment refer to colorectal and pancreatic cancers. Bayet-Robert et al. [64] performed a clinical phase I dose escalation trial of combination docetaxel chemotherapy with curcumin in advanced and metastatic breast cancer patients. The authors confirmed the safety profile of this combination therapy which was consistent with that observed with a monotherapy of docetaxel. Additionally, curcumin/docetaxel combination proved antitumour activity and a superior response rate in comparison to docetaxel in monotherapy. The recommended dose of curcumin is 6.0 g/day for seven consecutive days every 3 weeks in combination with a standard dose of docetaxel which proved its feasibility, safety, and tolerability. However, some scientific evidence demonstrated that dietary curcumin can inhibit chemotherapy-induced apoptosis in models of human breast cancer lines (MCF-7, MDA-MB-231, and BT-474) [314]. The chemotherapeutic agents evaluated were camptothecin, mechlorethamine, and doxorubicin-induced apoptosis. In conclusion, additional clinical studies are required to demonstrate the avoidance of curcumin (in both supplements and intake foods containing curcuma) in breast cancer patients undergoing chemotherapy.

In addition, this phytotherapeutic agent is well tolerated in human subjects. Therefore, curcumin could be considered an alternative nontoxic agent in the treatment of one of the most aggressive breast cancer, that is, triple negative breast cancer (ER-negative, PR-negative, and HER2/neu not over expressed) [303]. This breast cancer remains the most challenging factor in cancer treatment.

### 3.8. Green Tea

Green tea extract is prepared from the steamed and dried leaves of* Camellia sinensis* and contains flavonoids, a large group of polyphenolic compounds with antioxidants properties [269]. Epigallocatechin-3-gallate (EGCG) is the most abundant polyphenol in green tea and has been the focus of preclinical and clinical research on health beneficial effects [3]. However, the main mechanism by which green tea might help to prevent cancer has not been recognized.

*In vitro* and animal studies demonstrated that tea polyphenols can inhibit tumour cell proliferation and induce apoptosis [315, 316]. Additionally, tea catechins have revealed the ability to inhibit angiogenesis and tumour cell invasiveness as well as modulate the immune system function [317].

The chemopreventive potential of green tea contrasts with the consistent results from animal models. Evidence of green tea consumption on breast cancer prevention and development is not supported by epidemiologic studies and the role of green tea consumption in breast cancer remains unclear. The results of antiproliferative effect of green tea extracts or its polyphenols from human studies are inconsistent and depend on the type of cancer [318]. A systematic review and meta-analysis of prospective observational studies, 57 relevant articles, concluded that tea consumption has no significant effect on the risk of common malignancies including breast cancer [65]. A prospective cohort study performed in Japan found no association between green tea drinking and risk of breast cancer [66]. However, in a case-control study conducted by Zhang and colleagues in Southeast Chine between 2004 and 2005, despite the fact that the authors concluded that regular consumption of green tea can protect against breast cancer, they also suggested more research to closely examine the relationship between tea consumption and breast cancer risk [67].

In a follow-up study, despite prevention recurrence of stage I and II breast cancer being observed with an increase green tea intake, no improvements were confirmed in patients with stage III breast cancer. A potential prevention of green tea consumption in breast cancer recurrence in early-stage (I and II) cancer was also reported by Seely et al. [77].

In order to better evaluate the* in vivo* exposure to specific tea catechins, two studies incorporated prediagnostic biomarkers of green tea intake and metabolism on risk of breast cancer [68, 69]. In a prospective cohort in China, urinary tea catechins and their metabolites were measured in 353 cases and 701 controls and no association was found between urinary concentrations of biomarkers measured and risk of breast cancer [69]. Similar results were achieved in a prospective cohort Japanese study in which tea catechins biomarkers concentrations were measured in plasma [68]. In both studies, the detectable rates of some biomarkers were as low as 20–30%, which increased concern about the sensitivity of the assays, because ~50% of study participants reported drinking at least one cup of green tea daily. Crew et al. conducted a study using archived blood/urine from a phase IB randomized, placebo-controlled dose escalation trial of an oral green tea extract, polyphenon E (Poly E), in breast cancer patients [70]. The results suggested that the consumption of EGCG can have a preventive effect in breast cancer by influencing the growth factor signalling, angiogenesis, and lipid metabolism mechanisms.

In a cross-sectional study, including 3315 Asian women, daily green tea consumption demonstrated a significantly lower mammographic density percentage compared to nontea drinkers [319]. Mammographic density is a well-established breast cancer risk factor. The difference in mammographic density was observed mainly among postmenopausal women. The authors suggest that long-term exposure to green tea may act as a protective approach in breast cancer.

In addition, genetic factors may have an important role in the influence of green tea on breast cancer, namely, genetic polymorphism in angiotensin-converting enzyme gene and in the catechol-*O*-methyltransferase gene, probably due to the interindividual differences in the metabolism and elimination of tea polyphenols [71, 72]. In the specific case of catechol-*O*-methyltransferase gene, studies have inconsistent findings. Wu et al. [73] conducted a population-based case-control study in Asians in Los Angeles County and reported that consumption of green tea was associated with significant reduced risk of breast cancer in women carrying at least one copy of the low-activity catechol-*O*-methyltransferase allele relative to nondrinkers. In women carrying both high-activity catechol-*O*-methyltransferase alleles no association was found. In the Chinese population, the catechol-*O*-methyltransferase genotype did not present any modifying effect on the association between tea consumption and breast cancer risk.

Green tea has also demonstrated a promising role as adjuvant of chemo/radiotherapy due to both additive or synergistic effects and amelioration of cancer therapy side effects [320]. However, further clinical research is required to ascertain the effectiveness of these actions. EGCG can modify the pharmacokinetics of tamoxifen and induce chemosensitization in tamoxifen-resistant breast cancer cells [321]. In another study, the cotreatment of EGCG and tamoxifen increased apoptosis and reduced tumour growth in breast cancer cells using a murine model of breast carcinoma, enhancing the cytotoxicity of paclitaxel [322]. EGCG also has antiproliferation activity against estrogen-induced breast cancer cells (e.g., sunitinib) [323] and sensitives hormone responsive tumours to drugs that act in steroid receptors (e.g., tamoxifen) [321, 324]. Li et al. reported chemosensitization and synergistic anticancer effects with the coadministration of EGCG and histone deacetylase inhibitor trichostatin A in oestrogen receptor-negative breast cancer cells [325]. Zhang et al. conducted a clinical trial in breast cancer patients undergoing radiotherapy and supplemented with EGCG. The results showed that EGCG and its metabolites could potentiate the effects of radiotherapy [74]. Green tea also seems to protect the body against the harmful effects of radiation and chemotherapy [7, 320].

In the Minnesota Green Tea Trial, 1075 postmenopausal women at high risk of breast cancer due to dense breast tissue randomly consumed green tea extract (845 mg EGCG) or placebo, daily for one year. The safety of green tea was also tested. The main conclusion was that there were no statistically significant differences between groups in frequencies adverse events or serious adverse events, but EGCG consumption leads to a higher incidence of nausea, dermatologic events, and alanine aminotransferase elevation [75].

Lazzeroni et al. [76] studied the EGCG tissue distribution and evaluated its effect on cell proliferation in breast cancer patients. The consumption of 300 g of tea catechin extract phytosomes (equivalent to 44.9 mg of EGCG) increased the bioavailability of EGCG, which was detectable in breast tumour tissue and is associated with a decrease in the tumour circulating biomarker revealing antiproliferative effects on breast cancer tissue.

Based on the current data, large randomized intervention trials focusing on the efficacy of green tea polyphenols are required before a recommendation as preventive-cancer should be made.

No known contradictions are reported. Green tea has been consumed safely over thousands of years; recently a liver toxicity has been reported. However, this is probably related to the presence of contaminants in the plant.

### 3.9. Ginseng

The generic term ginseng encloses several species of plants belonging to the genus* Panax *such as* Panax ginseng* and* Panax japonicus* (i.e., Asian ginseng) and* Panax quinquefolius* L. (American ginseng) [269]. In recent years, ginseng has gained popularity in Western countries and is included in the Pharmacopoeias of Germany, Austria, and United Kingdom [326]; in the United States, ginseng is the second top-selling herbal supplement but it is not a drug approved by the Food and Drug Administration [327–329]. Ginseng presents a complex mixture of various active compounds but the main pharmacologically active ingredients are triterpene saponins known as ginsenosides, which are found in the roots. Therefore, the dried roots are used in traditional medicines due to the variety of beneficial effects, including in breast cancer [330, 331]. However, its clinical significance in breast cancer patients has not been fully investigated and some divergences are reported.

Despite several* in vitro* studies having proved the promising use of ginseng extract or its active components as anticancer agent in breast cancer [332, 333], no animal studies have been found in literature. The mechanisms by which components of ginseng or metabolites performed their antiproliferative effect are reported in several research studies and resumed in a recent review paper [334]. These compounds can modulate signalling pathways associated with inflammation, oxidative stress, angiogenesis, metastasis, and stem/progenitor-like properties of cancer cells. For example, ginsenoside Rp 1 inhibits the insulin-like growth factor 1 receptor (IGF-1R)/Akt pathway in breast cancer cells [332]. In addition, ginsenoside Rp 1 was also demonstrated to induce cycle arrest and apoptosis. Kwak et al. studied the inhibitory effect of ginseng sapogenins and their derivatives on the proliferation of MDA-MB-231 human breast cancer cells (a model of triple negative breast cancer) [333]. 20(*S*)-Protopanaxadiol exhibited IC_50_ (i.e., half maximal inhibitory concentration) comparable to the taxol (chemotherapeutic agent) and acts by stimulating caspase-dependent apoptosis in breast cancer cells. The ability of ginsenoside Rg 3, one of the major active compounds of heat-processed ginseng, to induce apoptosis in MDA-MB 231 cells by blocking NF- *ƙ*B signalling was also verified [335, 336].

A specific effect of ginseng in cancer is increasing the sensitivity of breast cancer cells to various chemical anticancer agents including gemcitabine (an antimetabolite), cisplatin (an alkylating agent), paclitaxel (a taxane agent belonging to a plant alkaloid), and epirubicin (an antibiotics) through downregulation of them RNA level of MDR-1 [337].

Despite popular use of ginseng in cancer patients, only a few clinical studies have been conducted on ginseng-chemotherapeutic agent association. A clinical phase II study using no ginseng alone but in Shengmai formula (i.e., a traditional Chinese ginseng preparation that contains red ginseng, lilyturf root, and magnolia vine fruit) reported immunologic improvements among breast cancer patients [78].

Some beneficial effects related to the use of ginseng in human include maintenance of natural energy, improvements of physical, chemical, and biological performance and enhancement mood and general vitality and immune function [326, 338]. Despite these positive outcomes which are attributed to its “adaptogen” characteristic, findings on the effects of ginseng in breast cancer patients are mixed. Bao et al. [79] conducted the Shanghai Breast Cancer Survival Study to detect some association between quality of life and postdiagnosis ginseng use among breast cancer survivors. The authors did not find any improvements. In another study, Cui and collaborators reported that the use of ginseng had positive quality of life scores, namely, in the psychological and social domains [80]. The authors explained the variability in response to the design of study and the different doses of ginseng use among breast cancer survivors.

Nevertheless, evidence of efficacy is sparse. Well-designed clinical trials are required to provide information for scientists and healthcare consumers. Furthermore, treatment of symptoms and side effects is crucial for people with cancer because of the longevity associated with successful cancer treatment. And regarding this issue, evidence is also required in relation to ginseng use.

Ginseng should be avoided by children and used with some prudence by patients medicated with blood pressure medicines, blood-thinning medications, hormones, or insulin due to possible drug-herb interactions (recommendation performed by American Cancer Society) [3]. Ginseng is relatively nontoxic but in high doses (i.e., superior to 3 g ginseng root daily) can confer adverse symptoms such as insomnia, nervous excitation, headaches, and nausea. Ginseng may present steroid/hormone like effects, so in women who have breast or endometrial cancer special attention to its use is recommended [7, 339].

### 3.10. Black Cohosh

Black cohosh, also known as* Cimicifuga racemosa* or* Actaea racemosa* (family of Ranunculaceae), is a popular phytotherapeutic product frequently used for women's health concerns such as premenstrual syndrome, dysmenorrhoea, and menopausal symptoms [3, 340]. A recent meta-analysis of nine controlled placebo clinical trial confirmed the efficacy of its use in relieving menopausal symptoms [341].

This plant is included in the famous patent medicine* Lydia Pinkham's Vegetable Compound* and was listed in the 19th century Pharmacopoeia [342]. Black cohosh contains unidentified substances with selective oestrogen receptor modulator properties; however, triterpenes glycosides have been assumed to be the crucial constituents for its biological effects [342].

Few* in vitro* tests using breast cancer cell lines in culture and* in vivo* animal studies evaluating the effect of black cohosh as chemopreventive or anticancer agents are reported in literature. Several components extracted from black cohosh were tested in human breast cancer cells revealing anticancer properties: cycloartane triterpenoids induced mitochondrial apoptosis and cell cycle arrest, via Raf/MEK/ERK signalling pathway and Akt phosphorylation [343] or via NF-*κ*B signalling pathway [344]. Actein revealed an antiangiogenic effect by inhibiting the proliferation and reduced the migration and motility of endothelial cells (*in vitro*). Oral administration of actein at 10 mg/kg for 7 days inhibited blood vessel formation and oral actein treatments (10–15 mg/kg) for 28 days resulted in decreasing mouse 4T1 breast tumour sizes and metastasis to lungs and liver [345]. Nevertheless, some contradictory conclusions have been indicated. For example, Einbond et al. conjugated a triterpene glycoside of black cohosh and actein to liposomes [346]. This vehicle increased the growth inhibition activity of actein against human breast cancer cells. Actein presented antiproliferative action by modulation of the NF-*ƙ*B and MEK pathways. Using female Sprague-Dawley rats, Weissenstein and colleagues indicated that black cohosh could be chemopreventive or chemotherapeutic agents for mammary cancer due to its immunohistochemistry effect [347]. However, Davis and collaborators suggested that black cohosh may increase metastatic mammary cancer in MMTV-neu mouse model which is used due to its similarities to HER2(+) breast cancer [348].

Black cohosh is one of the most controversial natural therapies used among breast cancer patients due to its ambiguous estrogenic or antiestrogenic activities with many studies in literature exploring considerable debate over the safety of its uses [349]. Under conditions of excessive estrogen, the active ingredients of this plant may behave as estrogen antagonists by a mechanism of competitive inhibition of the ER. However, in the presence of low estrogen, actives may act as weak agonists [350–352]. If black cohosh exhibits estrogenic activity, it may result in potentially negative outcomes on breast cancer risk or recurrence, mainly in women undergoing antiestrogen therapy [353]. However, Fritz and collaborators carried out a systematic review about the use of black cohosh in breast cancer and found that evidence is conflicting in all analysed aspects [81]. The authors concluded that current evidence does not sustain an association between black cohosh and increased risk of breast cancer (results from observational studies) and reduce evidence that supports the efficacy of black cohosh for reduction of hot flashes in breast cancer patients (results from observational studies and clinical trials). Some limitations of studies include subjective outcomes, different risk of bias, namely, lack of blinding and inadequate reporting of withdrawals (for clinical trials); variation of dose and duration schedules of black cohosh, different products and methods of extraction, and lack of information and criteria included in the retrospective design (for observational studies). In addition, black cohosh seems to have limited and no classic estrogenic activity as seen by its effect on bone metabolism.

Different conclusions have also been reported concerning the potential for interactions with antiproliferative effects of different classes of chemotherapy agents. A cohort study suggested that taking black cohosh can reduce risk of recurrence in patients taking tamoxifen [82]. No risk of recurrent and no consistent serious adverse events related to the combination of black cohosh and tamoxifen were reported in clinical trials [354, 355]. No interaction on the formestane- (i.e., an aromatase inhibitor-) induced tumour reduction was observed with the coadministration of black cohosh extract in a chemically induced rat model for mammary carcinoma [356]. In humans, different findings were reported [357, 358].

The Clinical Practice Guideline of the Canadian Society of Obstetricians and Gynaecologists list black cohosh interactions with some drugs including anesthetics, antihypertensives, and sedatives [359]. Despite this, Walji et al. conducted a systematic review and suggested that black cohosh has a high safety profile in cancer patients; however, the authors did not include recent evidence [360]. In another study based on animal studies, Freudenstein and colleagues suggested that* Cimicifuga racemosa* extract is safe for treatment of menopausal symptoms in breast cancer survivors in whom hormone-replacement therapy is contraindicated [361]. Case reports of hepatotoxicity have been reported but confounding factors such as “poor case data quality, uncertain of black cohosh product, quality, and insufficient adverse event definition” could justify this adverse effect [362].

The outcome of black cohosh uses in women with or without a history of breast cancer is unclear and its use must be discouraged.

### 3.11. Mistletoe

Mistletoe (*Viscum album* from the Viscaceae family), as part of anthroposophical medicine, is potentially effective against cancer and is used frequently in breast cancer due to its minimal side effects and the fact that these side effects are not life threatening [363]. The mistletoe contains different types of biological active ingredients, but the main constituents responsible for anticancer and immunomodulatory effects are lectins (ML-I, ML-II, and ML-III) [364, 365].

Experiments in cell cultures, animal models, and clinical data propose that cytotoxic and antitumour activities of mistletoe may be mediated by different mechanisms: apoptosis induction and necrosis, cell cycle inhibition [366, 367], and activation of specific and nonspecific immune system [368, 369].

Different* in vitro* studies demonstrated the antiproliferative effect of mistletoe extract against breast cancer cell lines [370, 371]. Kelter et al. proved that mistletoe extracts have cytotoxic activity on different human breast cancer cell lines and suggested that no growth stimulation of these cell occurred [370]. Using human breast carcinoma cell lines HCC 1937 and HCC 1143, Weissenstein and collaborators suggested that no herb-drug interactions occurred from the exposition of cancer cells simultaneously with doxorubicin (i.e., a chemotherapeutic) and* Viscum album* extract [371]. Additionally, at higher concentrations of mistletoe extract an additive* in vitro* inhibitory effect was observed. When* Viscum album* extract was associated with trastuzumab in an* in vitro* SK-BR-3 cells test, the results suggested no herb-drug interaction and exhibited a complementary anticancer effect [347]. A similar synergistic anticancer effect was observed in inhibition in the growth of both breast cancer cell lines (i.e., MCF-7—oestrogen receptor-positive—and MDA-MB 231-oestrogen receptor-negative) when the authors combined doxorubicin and lectin from Korean mistletoe [372]. Furthermore,* in vivo* investigations using different animal models were also presented in literature. For example, Beuth et al. reported the dose-dependent anticancer activity of mistletoe using a BALB/c mouse/BT474 ductal breast carcinoma model [373].

Several studies on breast cancer patients receiving chemotherapy report an efficacy on survival rate, tumour reduction and remission, and better quality of life with reduction of adverse reaction of standard chemotherapy when additionally treated with mistletoe products [84–87]. Safety and efficacy were set as the endpoints in a multicentric and comparative clinical trial conducted by Beuth et al. among women with primary breast cancer who received mistletoe extract [88]. In clinical trials, some limitations should also be pointed out such as limited sample size, lack of control, exclusion and inclusion criteria of the clinical trial, quality rating, and mistletoe preparations.

Twelve patients were selected by the presence of histological confirmed breast cancer tumour (≥2 cm in diameter) and included in a study to investigate the mistletoe effect in tumour regression of breast cancer. After six months, the mistletoe extract therapy demonstrated being highly effective [83].

Despite the promising results for the use of mistletoe in addition to chemotherapy, the discussion on the reduction of side effects and improvement of quality of life in breast cancer patients remains open and is still a controversial topic.

## 4. Nutritional Supplements

In cancer topic, three different phases could be passive of intervention with nutritional supplements: prevention, during conventional treatments after diagnosis and survival period.

Although studies have not established a specific role for vitamins and selenium in the prevention of breast cancer, some anticancer activities have been demonstrated using tumour cell lines (i.e.,* in vitro*) [374–376].

Some notable institutions in cancer research, such as the American Cancer Society, the World Cancer Research Fund, and American Institute for Cancer Research, advise against the use of nutritional supplements for cancer survivors [377, 378]. Nevertheless, the supplementation with multivitamins and minerals is frequent after a breast cancer diagnosis and in survivors who recognize them as anticancer and antioxidant agents [26, 34, 113, 379]. Despite this, the evidence base for nutritional supplementation in cancer patients during treatment remains inconsistent and ambiguous and the results obtained in some studies have been contradictory. For example, some observational studies performed in breast patients have not reported improvements in breast cancer prognostic [152, 380]; others showed beneficial effects [112, 123, 381] and some showed harmful events [123].

The information obtained with the studies that examine the association between supplementation use and cancer-related outcomes must be interpreted with care due to the methodological limitations of most study designs such as lack of complete prospective data on supplement uses, specifically around the time of prediagnosis, diagnosis, and treatment and lack of data collection on changes in supplement use over time. Greenlee et al. [379] published a prospective cohort study (the Pathways study) with methodological improvements over previous studies in which the authors provided specific detailed information on changes in supplementation use following diagnosis in a multiethnic population. In this study, most women used vitamin/mineral supplements before (84%) and after (82%) diagnosis. The most commonly initiated supplements were calcium and vitamin D; the most commonly discontinued supplements were multivitamins, vitamin C and vitamin E. In another study, the Intergroup Phase III Breast Cancer Chemotherapy trial (S0221), the authors collected data between 2003 and 2010 and reported that 48% of patients were taking multivitamins; 20% were taking vitamins C and D and omega 3 fatty acids in fish oils; 15% were taking vitamins E and B6 and folic acid; and 34% were taking calcium. In this study, the advice of clinicians related to the nutritional supplementation was diverse [382]. This review refers only to the most commonly used nutritional supplements among the breast cancer patients.

After reviewing the available scientific literature [157], at this moment no consensual recommendation for cancer patients is available even among the clinicians and a greater understanding of processes involved in the regulation of tumour growth is desirable.

### 4.1. Multivitamins

Generally, cancer patients have an augmented requirement for essential nutrients (e.g., vitamins, trace elements, and minerals) adequate levels of which are achieved with the supplementation products. This is particularly true before or during cancer destructive therapies for supporting their side effects better.

However, multivitamin supplements are usually a heterogeneous group of products with no standard composition that depends on the manufacturer, year of production, and batches [104, 106]. In the Swedish Mammography prospective cohort study, Larsson and collaborators highlighted an increase in the risk of developing breast cancer both for high frequency of consumption (19%) and for long duration of multivitamin supplementation (22%) [105].

Until now, no randomized trials have evaluated the outcomes of multivitamin supplementation on the toxicity or survival rate after breast cancer diagnostic [383]. However, Kwan and collaborators conducted an observational study in which 72% of women with early-stage breast cancer were self-prescribing multivitamins and reported neither beneficial nor harmful effects of these supplements on toxicity or survival [113]. Similar conclusions were found by Wassertheil-Smoller and collaborators in US postmenopausal women with invasive breast cancer [111]. However, other authors did not find any association of consumption of multivitamins and breast cancer risk [106, 107].

### 4.2. Antioxidant Vitamins and Minerals

There is scientific documentation that relates the high intake of antioxidant with both a lower risk of developing breast cancer [104, 110] and a positive impact in the mortality rates of cancer. In accordance with the American Cancer Society and Cancer Research UK, although the studies of nutritional supplements to reduce cancer risk have not all been disappointing, until now there is no consistent evidence that any type of nutritional supplement can help to prevent cancer, in contrast with the nutrients (including antioxidant) obtained in a healthy and balanced diet with abundance of fruits and vegetables [108, 384, 385]. Therefore, according to the American Cancer Society, the best advice is to get antioxidants through food sources rather than supplements.

The use of antioxidant agents in patients with cancer seems to be an intelligent idea based on their biologic mechanism, first because of their potential anticancer properties—that is, diminished oxidative damage; reduced proliferation and angiogenesis; increased apoptosis [386]—and second because they may reduce the oxidative damage from conventional cancer treatments involving chemo- and radiotherapy and therefore limited the toxicity of these therapies [383].

Despite the potential improvement outcomes, the supplementation with antioxidant agents (e.g., vitamin A, vitamin E, vitamin C, and selenium) during cancer treatment is discussed controversially mainly due to the probable interaction with or modification in the effects of conventional cancer treatments [386, 387]. Since radiotherapy and several chemotherapy agents (e.g., alkylating agents, anthracyclines, podophyllin derivatives, platinum complexes, and camptothecins) exert their anticancer properties through production of reactive oxygen species (ROS) and promoting apoptosis, the antioxidant agents may reduce the efficacy of radio- and chemotherapy-related cytotoxicity and consequently act as potential cancer-promoting. Antioxidant supplements appear to successfully block otherwise effective prooxidant therapies and protect both normal and tumour cells from the oxidative damage [106]. In this context, some studies highlight the adverse effects of antioxidant supplementation on overall mortality for patients with cancer [388, 389]. However, other studies proved the benefits of antioxidant supplementation in a specific treatment (e.g., chemotherapy [112]; radiotherapy [390]; and both [381]). Based on these restricted outcomes of the observational studies and clinical trials, there does not appear to be obvious evidence concerning the effect of antioxidant supplementation and its use during chemo and radiation treatments. Therefore, high-quality placebo-controlled trials are needed.

#### 4.2.1. Vitamin A and Carotenoids

Vitamin A refers to a group of compounds named retinoids which cooperate in a large variety of physiological processes such as in vision, bone growth, reproduction, cell division, and differentiation [391, 392]. Two forms of vitamin A can be ingested via diet: preformed vitamin A, found in foods derived from animal sources (e.g., liver, whole milk) and absorbed as retinol, and provitamin A carotenoids, derived from fruits and green leafy vegetables and converted into retinol once ingested. Most of the supplements contain the preformed vitamin A [391]. It is stored in the liver. Synthetic retinoids are also available such as bexarotene and fenretinide.

Various longitudinal cohort studies, performed in different ethnical groups and geographic locations worldwide, evaluated the intake of carotenoid and endogenous retinol levels with the risk for developing breast cancer [115–118]. The type of beneficial carotenoids is controversial [115–121]. For example, in the postmenopausal women population, some studies did not correlate the retinol levels with breast cancer risk [115, 117]. Other studies demonstrated different effects between the lycopene levels (i.e., a carotenoid substance that does not convert into vitamin A) and the risk of breast cancer, that is, an increased risk [116, 119] or a protective effect among ER-positive and progesterone receptor-positive breast cancer [120].

The European Prospective Investigation into Cancer and Nutrition cohort studied 1502 female incident breast cancer cases (premenopausal (*n* = 582) and oestrogen receptor-negative cases (*n* = 462)). Carotenoids, tocopherols, vitamin C, and retinol plasma levels were determined to find an association with risk of breast cancer. The results showed that a higher concentration of plasma *β*-carotene and *α*-carotene is associated with lower breast cancer risk of oestrogen receptor-negative tumours and higher risk of breast cancer was found for retinol in relation to oestrogen receptor-negative/progesterone receptor-negative tumours. There was no statistical difference for the other studied compounds [121].

A positive relationship between a high plasma carotenoids and breast cancer survivals was reported by Rock et al. in the Women's Healthy Eating and Living study [122].

Higher biological exposure to carotenoids, when assessed over the period of the study, was associated with greater likelihood of breast cancer-free survival regardless of study group assignment.

#### 4.2.2. Vitamin C

Vitamin C, or ascorbic acid, is an essential water-soluble vitamin that acts as antioxidant and has important biological roles such as in protein metabolism, including the biosynthesis of collagen, neurotransmitters, and L-carnitine; in immune function and in absorption of iron from plant-derived foods [391]. This vitamin, which is crucial for the structural integrity of intercellular matrix, is produced by the most animals but not by humans who must get it from the diet or as supplement.

There is restricted evidence of using vitamin C supplementation in the primary prevention or delay of total cancer incidence, including breast cancer [115, 393]. One of the largest studies in women, followed up for 9.4 years, reported that the supplementation with 500 mg daily of vitamin C had no effect on the occurrence of breast cancer [393]. However, in a cohort study including postmenopausal women, Cui and colleagues found a significant increased risk of breast cancer with high dose of vitamin C supplementation [120].

The safety of oral vitamin C supplements subsequent of the cancer diagnosis is not obvious [386]. The attention given to vitamin C is increasing since the publication of the* in vitro* study by Chen and collaborators [394] which verified the selective apoptosis of cancer cells induced by high concentrations of vitamin C. This effect was also supported by Ullah et al. [395]. Additionally, vitamin C enhances immunity and presents antioxidant properties including the neutralization of free radicals which may interfere with cancer progression [396]. The important issue is if these beneficial outcomes can be effective* in vivo* (i.e., in human body) considering the solubility of this vitamin and some parameters should be clarified, namely, the dose of vitamin C, the timing of supplementation, the side effects of high concentration of vitamin C (e.g., for kidneys), and its effect in combination with pharmacological and conventional cancer therapies (e.g., chemo- and radiotherapy). These properties are controversial and seem to be dependent on the dose, the source of vitamin C intake (i.e., derived from food or supplementation), and the timing and duration of intake [125, 397]. For example, some studies associated the dietary vitamin C intake with reduced risk of breast cancer mortality [125, 398] and no relationship demonstrated in other studies [26]. Additionally, the results also varied in the case of vitamin C supplementation. Studies reported inverse association between vitamin C supplementation, most of them referred to postdiagnosis breast cancer supplementation and mortality or recurrence [123, 126, 381], and no association was reported by Harris et al. [125]; however, this study presented a limited power analysis. These differences are probably related to the limitations of each study (i.e., small population with no confidence intervals or statistical analysis; details of concurrent treatment, heterogeneity across included studies). The relationship between vitamin C supplement intake and breast cancer risk was evaluated in an epidemiologic study with 57,403 postmenopausal women via food-frequency and supplement questionnaires. Vitamin C supplement was not associated with breast cancer risk overall but was associated with increased postmenopausal breast cancer risk in women with high vitamin C intake from foods [124].

Concerning the use of antioxidant supplements, including vitamin C, during conventional treatment of cancer, the evidence from experimental studies and observational or clinical trials is also controversial. Jacobs et al., since there is no high-quality evidence to confirm the benefits of vitamin supplementation in cancer patients (either increases the antitumour effects of chemotherapy or reduces its toxicity), do not recommend the use of this vitamin until double-blind placebo-controlled trials are completed [399]. Moreover, Subramani and collaborators verified that the pretreatment of MCF-7 breast cancer cells with vitamin C, in a dose-dependent reply, protected them against lipid peroxidation caused by tamoxifen treatment [400]. However, Hubner and Hanf suggested that the vitamin C from dietary sources does not have negative effects not only in chemo- and radiotherapy but also for targeted drugs [397]. Vitamin C (500 mg daily) supplementation in combination with vitamin E (400 mg daily) and tamoxifen therapy, for the period of 3 months, in postmenopausal women with breast cancer reduced the tamoxifen effect in plasma lipid and lipoprotein levels [127]. The tamoxifen therapy may enhance the synthesis of VLDL and diminish the activity of lipoprotein lipase which hydrolyses triglycerides [391]. A retrospective study showed fewer side effects of chemotherapy in breast cancer patients supplemented with low-dose infusion of vitamin C [401]; nonetheless this study did not refer to recurrence and survival data and conclusions about its safety could not be assessed. In a randomized 5-month study, Suhail and collaborators concluded that the supplementation of vitamin C (500 mg daily) and vitamin E (400 mg daily) restores antioxidant status, lowered by the breast cancer and chemotherapy, and reduces the DNA damage [128]. The authors also suggested that this regimen of supplementation should be helpful in protecting against the side effects associated with the cycles of chemotherapy treatments. Other studies reported similar conclusions after intravenous vitamin C administration [129, 130]. For example, Vollbracht et al. conducted a retrospective, multicentre, epidemiological cohort study which proved that the intravenous vitamin C administration improves quality of life in breast cancer patients during chemo/radiotherapy and aftercare [130]. In this context, the route (oral versus intravenous) used for vitamin C supplementation should also be considered when evaluating the efficacy and safety among cancer patients. Pharmacokinetic studies suggest that much higher levels of plasmatic vitamin C can be achieved by bypassing the oral route [402].

The dose of vitamin C supplementation varied in the breast cancer patients from 400 mg or less per day (in the Shanghai Breast Cancer Survival Study [381]) to higher than 1 g [403]. Development validated randomized trials are warranted to define if these higher amounts are safe and which dosage is required to reach the experimental concentrations described by Chen et al. [394]. Different levels of intake (from both dietary and supplementation) may influence the safety and efficacy in cancer patients [386]. Hoffer et al., in a dose-finding phase I study, demonstrated that the intravenous administration of ascorbic acid in a low-dose had inferior outcomes compared to patients supplemented with higher doses [404].

Ascorbic acid is a critical nutrient for the synthesis and integrity of collagen and for the optimal stability of the extracellular matrix which are essential factors for controlling cancer. Based on the presumption that cancer patients have low reserves of ascorbic acid [405], Cha and colleagues showed that the supplementation of ascorbate in ascorbate-restricted mice injected with breast cancer cells reduced tumour growth and enhanced encapsulation of tumours [406]. Additionally, it modulated inflammatory cytokine secretion. These results support the proposed approach of using vitamin C to treat the cancer [407]. The administration of intratumoural vitamin C delayed tumour growth in murine solid tumour models and synergistic antitumour effects were observed with cisplatin [408]. However, this study was performed on animals. So, the use of vitamin C as anticancer therapy is not recommended in cancer patients.

#### 4.2.3. Vitamin E

Vitamin E is a liposoluble vitamin that exhibits different pharmacological properties such as antioxidant, anti-inflammatory, and inhibition of protein kinase C [391]. It can be acquired from some dietary sources (e.g., nuts, seeds, vegetal oils, green leafy vegetables, and fortified cereals) or as a supplement. Among its different chemical forms, the alpha-tocopherol is the main and most active form achieved in human plasma and studied in clinical trials.

The supplementation with vitamin E in breast cancer patients has also different outcomes. Some of the effects of vitamin E in breast cancer have been explored previously with the coadministration of other antioxidant vitamins (e.g., vitamin C). Other studies have shown that long-term uptake of vitamin E could have a negative effect on breast cancer patients [114, 131]. The HOPE-TOO trial revealed no effects of long-term vitamin E supplementation (7.1 years) on individual rates of breast cancer [409]. Nagel and collaborators did not find any association between long-term dietary intake vitamin E (8.8 years) and risk of breast cancer development [109]. Alpha-tocopherol acetate (400 mg) supplementation increased biomarkers of estrogen-stimulation in 5 out of 7 breast cancer patients while taking tamoxifen suggesting that vitamin E supplements may decrease the antiproliferative effect of tamoxifen [132].

Tam et al. verified that alpha-tocopheryl succinate, a synthetic derivative of alpha-tocopherol, improved the cells' sensitivity to doxorubicin (anticancer agent) which reduced the cell viability in cancerous breast tissue samples [410]. This combination, using vitamin E or its analogue in a supplementation regimen, is promising for the treatment of cancer. Random placebo-controlled trials showed that the association of pentoxifylline and vitamin E after radiotherapy in breast cancer women may be used to prevent radiation-induced side effects [133, 134].

In another study, the intracardiac injection of Trolox inhibited osteolysis bone metastasis caused by breast cancer in an experimental metastasis model [411]. Despite this, vitamin E analogue did not have any effect in the mammary fat pad model; it suppressed breast cancer cell-induced osteoclast differentiation and the invasive behaviour of cancer cells via prostaglandin E2- (PGE2-) dependent and PGE2-independent mechanisms.

#### 4.2.4. Selenium

Selenium is an antioxidant mineral that activates enzymes (e.g., glutathione peroxidase) which participate in the metabolism of oxidants and drugs [412]. However, this activation is dependent on the physiological selenium concentrations which should be between 70 and 90 mcg/L [413]. In humans, physiological selenium concentrations depend on the intake of food products containing high levels of selenium (e.g., grains, cereals, organ meats, and seafood, with lower amounts in dairy products, fruits, and vegetables), the selenium content in soil of each geographic region, and the supplementation [397]. However different organic nutritional forms of selenium are available for cancer prevention; sodium selenite is the favourite form of selenium for therapeutic purposes [414].

Selenium appears to be a crucial trace element recognized in some types of cancers as cancer-protective agent [415]. Adequate selenium levels should be maintained to provide therapeutic benefits such as preventive activity in breast cancer [416]. In a meta-analysis, prospective studies demonstrated the protective effect in cancer incidence when patients were supplemented with selenium in the case of deficiency in physiological levels [417]. Nevertheless, the results from studies are again unclear. In another meta-analysis study, the authors evaluated the association between selenium exposure/supplement and cancer risk and did not find a protective efficacy of selenium supplement [418]. Additionally, different effects (i.e., decreased or not associated effect) on specific types of cancer were reported; namely, it decreased the risk of breast cancer. In a review paper related to the prevention of cancer by selenium, the authors reported that positive evidence was only achieved from epidemiological data and not from randomized studies [415]. From this perspective, before the supplementation of cancer patients with selenium (e.g., sodium selenite), the individual selenium status should be measured (e.g., selenium in whole blood) [419] to avoid overdosing and side effects such as higher incidence of serum lipids, hypertension, and diabetes [391].

To investigate the input of selenium and other trace elements in the etiology of breast cancer, Adeoti and collaborators determined the serum concentration of these elements in breast cancer patients [420]. An inverse relationship between the concentration of zinc and selenium in the venous blood was verified while that of the control reported a direct relationship. The authors demonstrated the association between the serum concentration of trace elements, including selenium, and breast cancer.

In addition, selenium seems to reduce the side effects of radiotherapy and does not affect the efficacy of conventional treatments [421]. In this study, diarrhoea was significantly reduced in the group supplemented with selenium.

#### 4.2.5. Vitamin D and Calcium

Vitamin D is a liposoluble vitamin mainly acquired through endogenous synthesis via sun exposure of the skin (ultraviolet B rays); a daily sunlight exposure can generate the equivalent of a 10,000 IU oral dose of vitamin D3 [188]. It can also be obtained from dietary sources (e.g., fatty fish and fortified food products, cereal, milk and dairy products, beef, and liver) or as a nutritional supplement (ergocalciferol (D2) or cholecalciferol (D3)) [422]. Either of these forms needs to be metabolized via hydroxylation in the liver and kidney to the active form known as calcitriol. Our levels of vitamin D are mainly affected by the limited sun exposure (darker skin, use of sunscreen, season, latitude, and time of day) and limited physical activity. However, daily recommended intake (DRI) values generally consider vitamin D levels in persons with limited sun exposure as adequate to restore levels. Several other biological systems (e.g., heart, brain, muscle, immune, pancreas, and control cell cycle) present vitamin D receptors. Physiologically, appropriate levels of vitamin D are essential in skeletal mineralization, regulation of parathyroid hormone production, and maintenance of calcium and phosphorous plasmatic concentrations [423]. Vitamin D regulates intestinal calcium absorption and bone and renal calcium resorption [422]. Regarding the topic of cancer, it presents promising actions [424]: regulating the expression of genes that are involved in development and progression of cancer; being able to stimulate cell differentiation and apoptosis; inhibiting proliferation, angiogenesis, invasion, inflammation, and metastatic potential; suppressing aromatase activity leading to reduce estrogen levels and reduced breast cancer risk. Blood levels of 25-hydroxy-vitamin D can be measured to determine a deficiency of vitamin D [423].

Vitamin D deficiency is common among cancer patients, namely, in breast cancer cases [135, 154, 160, 167, 170, 425]. Some authors found no association between breast cancer risk and vitamin D/calcium intake [136, 138–140, 142] or serum levels [145–147]. Studies about intake or serum vitamin D and/or calcium levels found controversial results: dietary vitamin D or serum levels were associated with a decrease of breast cancer risk in several countries such as Pakistan [154], Iran [161], Korea [162], USA [163, 164], Europe [165], Australia [156], France [150], Italy [153], and Germany [166]; dietary calcium and vitamin D had no association with breast cancer risk in a large cohort study performed in Europe [141] but other authors found a significant evidence of the inverse relationship between vitamin D and calcium intake and breast cancer risk, related or not to menopausal status [157, 158], a U-shape association between plasma vitamin D levels and breast cancer risk while an inverse association was observed with serum calcium levels [171–173]. Serum calcium levels were inversely associated with breast cancer in premenopausal women and the opposite occurred in overweight postmenopausal women [169]. However, in Asian population calcium serum levels and breast cancer were not related [148]. The risk of breast cancer also appears to vary with menopausal status. While no relation between vitamin D serum levels was found in premenopausal women, an inverse association seems to be evident in postmenopause with threshold serum values of 27 ng/ml [168]. Lee et al. found that vitamin D has a protective effect on premenopausal women [155]. Despite the fact that age may be a risk factor, Mohr et al. showed no relationship between vitamin D levels and breast cancer in young military women [137], and results in postmenopausal women revealed no association either [136, 145]. Until now there is no agreement about the optimal dietary vitamin D and calcium intake to diminish breast cancer risk. However, based in existing studies, some authors suggested that daily intake of 600 mg calcium + 400 IU vitamin D and a target of 30–50 ng/ml of serum vitamin D may achieve the lowest risk of breast cancer in women [153, 166, 426].

Discordant data about the dietary source of those nutrients have been also discussed. Dietary but not vitamin D supplement was positively associated with increased breast cancer risk [176]. Other studies found no association with dairy products consume and breast cancer risk [149, 151, 427]. Sun exposure is also a relevant source of vitamin D and it seems to be an important protective factor when combined with dietary vitamin D, especially in postmenopausal women at northern latitudes with poor UV light [152].

Nonetheless, there are no adequate clinical trials that support the promising outcomes of supplementation breast cancer patients, in the case of a shortfall, with vitamin D. Most of the studies referred to the fact that the results required cautious interpretations. In accordance with Goodwin et al. [181], vitamin D deficiency in these patients is a negative prognostic factor. Women with aggressive subtypes of breast cancer have lower serum vitamin D levels which can be an indicator of poor prognostic [177, 183, 184]. For example, in a multiethnic cohort, Villaseñor and collaborators revealed that higher serum of 25-hydroxy vitamin D may be associated with improved survival, but the results were not statistically significant, referring to the need of including additional endpoints in future larger studies [182]. Calcium serum levels were positively related to breast cancer aggressiveness in premenopausal women with or without overweight [185]. The calcium/magnesium ratio appears to be also important since they have antagonist effects on absorption-resorption cycle. Magnesium intake has been related to an improved breast cancer survival and this effect is potentiated with higher calcium/magnesium ratios [180].

Vitamin D intake was not related to breast cancer recurrence independently of menopausal status [179].

Some studies related the lower risk of breast cancer and vitamin D to the inhibition of cell proliferation via nuclear vitamin D receptor (VDR). Polymorphism of VDR may be determinant in the breast cancer risk and may also explain the controversial data from different epidemiologic studies [176, 178, 428]. However, despite contradictory results reported in different studies, polymorphism of this receptor has been pointed as responsible for individual sensitivity to vitamin D [174, 429]. This relation might be also dependent on breast cancer subtype and menopausal status [175].

As with other nutritional supplements,* in vitro* and* in vivo* studies using vitamin D in breast cancer patients demonstrated contradictory results. Because a high concentration of calcitriol induces the hormone transcriptional targets and presents antiproliferative effects in culture breast cancer lineages, Urata et al. evaluated the outcomes of calcitriol supplementation in postmenopausal breast cancer specimens [430]. However, the authors could not extrapolate the effects observed* in vitro* to* in vivo* analyses.

In this context, a universal benefit from vitamin D supplementation among patients with cancer will be influenced by various factors, including the baseline vitamin D status, vitamin D receptor polymorphisms, and variable target effects dependent on the vitamin D receptor status of the tumour [383].

Many breast cancer patients have different risk factors for the development of osteoporosis such as age or aromatase inhibitor therapy. The use of aromatase inhibitor in postmenopausal women can generate a harmful effect on bone mass and an augmentation in fracture risks. These musculoskeletal symptoms appear probably due to estrogen deficiency caused by aromatase inhibitor [431]. Vitamin D is a promising and effective approach to reduce the incidence and severity of arthralgia resulting from aromatase inhibitor treatment [186, 190, 191, 193]. Sometimes, even with the supplementation of 500–1.000 UI women present a vitamin D deficit [186, 431]. Khan et al. [190] successfully supplemented patients with high doses of vitamin D (50,000 IU per week) for several weeks to control joint pain and fatigue associated with letrozole (i.e., an aromatase inhibitor). Other authors achieve similar improvement with calcium and/or vitamin D supplementation [187, 192, 432].

Amir and colleagues [188] explored the effects of high dose vitamin D3 (10,000 IU/day for 4 months) in breast cancer patients with bone metastases. In this phase 2 trial, the authors suggested the safety of supplementation but neither significant palliative benefit nor significant change in bone resorption occurred.

In breast cancer patients, whose bone density can be affected by chemotherapy-induce menopause and aromatase inhibitor, clinical practices guidelines recommend the supplementation not only with vitamin D but also with calcium [189]. Calcium is the most prevalent mineral in the body. Chung et al. reported no benefits for bone density or risk of fractures in breast cancer patients supplemented only with vitamin D and limited benefits for combination higher doses of vitamin D (>10 *μ*g/day) with calcium (>1,000 mg) in noninstitutionalized individuals [433]. In a systematic review, results from trials point to insufficient calcium and vitamin D supplementation doses (i.e., 500–1500 mg for calcium and 200–1000 IU vitamin D) to prevent bone mineral density loss [189]. Vitamin D supplementation was associated with an improvement in bone loss if target serum levels of 30 ng/ml were achieved [194], but some negative results are also reported. Doses of 500–1500 mg calcium and 200–1000 IU vitamin D were not sufficient to prevent bone mineral loss [189]. Some of the studies reported associated calcium supplementation with the enhanced risk of cardiovascular disease, so future validated trials should be considered to evaluate the safety and efficacy of these regimens of supplementation in women undergoing breast cancer therapy. Another aspect to consider is the confounding factor in human trials. Deschasaux et al. found that body mass index and alcohol consumption may modify the effect of vitamin D on breast cancer risk, which can be the reason for discrepant results between studies [159].

There are very few randomized placebo-controlled trials that proved the efficacy, safety, and optimum dosage of vitamin D and calcium supplementation for cancer patients. Rohan et al. found no benefit in the administration of daily use of 1000 mg of calcium carbonate and 400 IU of vitamin D3 for 7 years, in postmenopausal women and the risk of benign proliferative breast disease [143]. The same study concluded that daily doses used were not associated with a protective effect related to breast cancer risk [144].

Vitamin D deficiency has also been linked with increased toxicity from bisphosphonate therapy, which can provoke hypocalcaemia [434]. Currently, researchers investigate the need to supplement vitamin D and calcium simultaneously with bisphosphonate therapy [435]. In a double-blind, randomized, placebo-controlled study, Rhee et al. proved the efficacy of combined alendronate (5 mg) and calcitriol (0.5 *µ*g) to prevent bone loss due to aromatase inhibitor in Korean postmenopausal women with early breast cancer.

Despite the huge data from experimental and observational studies, a better understanding of the biologic effect of vitamin D in breast tissue and a more careful clinical design will be useful for making recommendations for vitamin D supplementation among breast cancer prevention or treatment.

#### 4.2.6. B Complex Vitamins

B complex vitamins include eight water-soluble vitamins: vitamin B_1_ (thiamine), vitamin B_2_ (riboflavin), vitamin B_3_ (niacin or niacinamide), vitamin B_5_ (pantothenic acid), vitamin B_6_ (pyridoxine), vitamin B_7_ (biotin), vitamin B_9_ (folic acid), and vitamin B_12_ (cobalamins; cyanocobalamin) [391]. Each B complex vitamin presents a specific function in the human organs. Some of them can be found naturally in unprocessed food (e.g., beans, meat, poultry, fish, eggs, milk, peas, select fruits, and vegetables) or fortified products (e.g., fortified cereals). Additionally, supplementation with complex B vitamins is also considered as an approach in the case of breast cancer patients or survivors, namely, folic acid [436]. These authors concluded that folic acid supplementation may promote the progression of established breast tumours.

Different conclusions were also reported in the literature related to the effect of B complex vitamins and the risk for breast cancer development [195–199, 201–203, 205–218, 437]. In different clinical studies, despite the fact that several authors verified that some vitamins of B complex (e.g., folate, vitamin B_6_, and vitamin B_12_) did not reduced the risk of developing breast cancer [202, 216, 437] even when stratified by hormone receptor status, other ones reported an association [195, 199, 208–213, 217]. For example, using data from the European Prospective Investigation into Cancer and Nutrition (EPIC), that is, a large prospective cohort study including 23 centres in 10 European countries [203], the plasma folate and vitamin B_12_ levels were not associated with the risk of breast cancer or by hormone receptor status [437]. Kim and colleagues indicated that high folate plasma concentrations may be associated with increased breast cancer risk among women with a* BRCA1/2* mutation (i.e., tumour suppressor genes) [195]. In contrast with these results, a higher dietary folate intake may diminish breast cancer risk and this association may differ by menopausal and sex hormone receptor status [213, 214]. In another based EPIC cohort study, the main conclusions were as follows: high vitamin B_6_ plasma concentrations may reduce the breast cancer risk, particularly of estrogen receptor (+) breast cancer; high riboflavin plasma levels may reduce the breast cancer risk in premenopausal but not in postmenopausal women; and homocysteine and the other B vitamins do not seem to influence breast cancer risk [218]. In a large randomized, controlled trials, combined B vitamins daily supplementation (vitamin B_6_, 50 mg; vitamin B12, 1 mg; folate, 2.5 mg), administrated over a period of 7.3 years, had no significant effect on the cancer breast risk [196]. In different meta-analysis studies concerning folate plasmatic levels or folate (from diet and/or supplementation), the authors reported no association between folate intake and risk for developing breast cancer [215], and this did not vary by menopausal status or hormonal receptor status [197, 198]. In addition, some of these studies suggested that adequate ingestion of folate may have protective effects against breast cancer risk in women with moderate to high alcohol consumption level [215]. Zhang et al. also achieved similar conclusions; that is, folate intake had little or no effect on the risk of breast cancer; moreover, a dose-response meta-analysis suggested an association between folate intake and breast cancer risk; daily folate intake of 200–320 *µ*g appeared to associate with a lower risk and a daily folate intake >400 *µ*g/d with an increased risk [205]. In a systematic review of clinical studies, Castillo and collaborators suggest a caution in women exposed to high folate intake during the folic acid fortification era, once some studies demonstrated a higher risk of this population for development breast cancer [201]. A weak relationship between the dietary vitamin B2 intake and the reduction of breast cancer risk was also shown in another systematic review and meta-analysis study [207].

Some vitamins of the B complex can interact with one-carbon metabolizing genes which can have an important role in the breast cancer development [219–224]. For example, some case-control studies assessed the association between MTHFR (5,10-methylenetetrahydrofolate reductase) and MTR (methionine synthase) genotypes and breast cancer risk [219–224]. These enzymes are involved in the metabolism of folate and homocysteine and their deficiencies could explain some alterations during breast carcinogenesis. The results proved that some MTHFR (e.g., C667T and 2756GG genotypes) and MTR polymorphisms (e.g., 2756GG genotype) are associated with risk of development breast cancer in different populations [219, 221–224]. Despite the fact that several studies reported an influence of dietary specific B complex vitamins (e.g., folate, vitamin B6, and vitamin B12) intakes on these associations [221–224], some authors stated no association [219, 220, 223, 224]. Dietary methyl group donors such as some B complex vitamins could influence the hypermethylation status of certain genes. Pirouzpanah and colleagues showed that individual B vitamins can present different effects on promoter hypermethylation and methylation-related expression of retinoic acid receptor-beta (RARB) and breast cancer-1 (BRCA1) genes in Iranian patients with breast cancer [225]. Hypermethylation at promoters of RARB and BRCA1 is associated with reduced transcript levels of the respective gene in primary breast cancer tissue samples.

The folate also plays an important role in the regenerating methionine, the methyl donor for methylation, and in the DNA synthesis and repair and, consequently, in carcinogenesis process [438]. In a case-control study involving patients at a tertiary hospital in Uganda, the red blood cell folate levels were not associated with breast cancer risk [204].

Concerning the influence of folate in survival, a prospective cohort study reported that folate supplementation is unlikely to have a significant adverse effect on breast cancer survival among women treated with chemotherapy [200]. In another case-control study, the authors verified that higher dietary vitamins B_1_ and B_3_ intake as well as specific polymorphisms of one-carbon metabolizing genes were associated with improved breast cancer survival [226].

Some chemotherapy often originates cutaneous side effects, namely, dry, itchy, and irritable skin due to nonspecific inhibition of the proliferative activity of keratinocytes. Based on the skin barrier stabilizing effect of vitamin B_3_ (niacinamide), Wohlrab et al. [227] conducted a multicentre prospective randomized reference-controlled crossover study and proved the superiority of topical preparation containing niacinamide compared to standard care. The authors demonstrated the cytoprotective and barrier stabilizing effect of vitamin B3 and its prophylactic application for controlling the cutaneous symptoms and maintaining quality of life in breast cancer patients while undergoing cytostatic therapy.

### 4.3. Omega 3 Polyunsaturated Fatty Acids (PUFA)

Eicosapentaenoic acid (EPA) and docosahexaenoic acid (DHA) are long-chain (*n*-3) polyunsaturated and highly peroxidizable fatty acids which are obtained mainly from marine sources. These supplements have important anti-inflammatory properties. Based on the results of preclinical and clinical studies, supplementation with omega 3 PUFA seems to be a promising approach among breast cancer patients. Food sources may be used with similar results [228]. Due to their nature, safety should not be a critical issue.

In a long-term prospective study, Brasky et al. showed an inverse association between the supplementation with omega-3 fatty acid and breast cancer risk [235]. This association depends on the type of fatty acids. For example, Pouchieu and colleagues [229] proved that specific plasma saturated, monounsaturated, and polyunsaturated fatty acids were differently associated with breast cancer risk. These authors reported that the total PUFAs were correlated with breast cancer risk only in the placebo group. Additionally, a modulation role of antioxidant agents in these associations via neutralizing the potential effects of these fatty acids on carcinogenesis was also suggested.

Different meta-analysis of epidemiological studies suggested that fish consumption and dietary fatty acids might be not associated with breast cancer risk [231–234]. Some authors proposed conducting well-designed prospective studies to explore the role of fish consumption/dietary fatty acids in relation to breast cancer risk [233]. However, Zheng et al. found an inverse relationship between dietary marine *n*-3 PUFA and breast cancer risk. The increment of dietary *n*-3 PUFA of 0,1 g/day may reduce breast cancer risk in 5% [238]. So, dietary oil fish intake or supplementation had a protective effect in breast cancer patients [236]. More pronounced preventive effects were found between omega 3 and postmenopausal women at risk [236, 237]. The populations who consume high levels of omega 3 and low levels of omega 6 showed a breast cancer risk reduction. In contrast to omega 3, omega 6 induces inflammation reactions [241]. In a meta-analysis, Yang et al. also demonstrated the negative association between the higher omega 3: omega 6 ratio intake and breast cancer risk [239]. Similar results were achieved by Murff and collaborators [240].

The protective effect of omega-3 fatty acids was putted to the test in a pilot study of 35 postmenopausal women with cytological evidence of hyperplasia, with an intake of 1860 mg EPA + 1500 mg DHA ethyl esters daily for 6 months. A favourable decrease in several breast cancer biomarkers has indicated the need of further placebo-controlled clinical trials [243].

Patterson and collaborators did not associate EPA and DHA intake from fish oil supplements with breast cancer outcomes [230]. Sandhu et al. found an increased effect of protective omega-3 fatty acid supplementation in higher body mass index women [244].

To determine the dose of omega-3 fatty acids that reach the maximal target tissue effects in women at high risk of breast cancer, Yee et al. [242] suggested that doses up to 7.56 g of DHA and EPA (per day) were well tolerated with optimal compliance. A combination of omega 3 (4 g) and raloxifene (30 mg)—a breast cancer chemopreventive agent)—was successful in reduction insulin-like growth factor (IGF-1) levels and omega 3 added the additional effects of improving serum lipids, antioxidant, and anti-inflammatory activities [245, 246].

EPA and DHA can increase the production of ROS in cancer cells, so they are being investigated as promising adjuvants of cancer treatment (e.g., chemotherapy; radiotherapy) to maximize the sensitivity of residual tumour cells to the therapy and maintain or (preferably) decrease the sensitivity on nontumour cells without any additional side effects [383, 439]. A phase II trial has proved the safety and feasibility benefits of omega 3 PUFA when supplemented together with chemotherapy. Bougnoux et al. [247] supplemented with DHA (1.8 g/day) metastatic breast cancer patients that were receiving anthracycline-based chemotherapy. Despite the limited number of patients (i.e., *n* = 25), the authors reported an increase disease-free survival and a longer time to progression in patients with high DHA incorporation into plasma phospholipids.

Considering the effects of EPA and DHA on cellular processes of bone turnover, these fatty oils may compensate aromatase inhibitors effects to bone and seems to be a promising approach in this clinical situation. Hutchins-Wiese et al. [248] supplemented postmenopausal breast cancer survivors with aromatase inhibitors with a high dose of DHA and EPA (4 g/daily for 3 months) and demonstrated that PUFA supplements can reduce bone resorption. Considering the short-term effects of fish oil supplementation, long-term studies are required.

## 5. Conclusions and Perspectives

Breast cancer is one of the most leading causes of cancer death among women. Women with a breast cancer history often resort to alternative and complementary therapies, mainly phytotherapeutic products and nutritional supplements, for the management of the typical symptoms and adverse effects of conventional cancer treatments. Although extensively used, these products are poorly regulated and can have either positive (e.g., synergetic effects) or negative (e.g., metabolic and drug interactions, diminishing the therapeutic benefits of conventional cancer treatments). There is a lack of high-quality scientific evidence for many of the most phytotherapeutic products and nutritional supplements and more clinical scientific evidences concerning the safety and efficacy are mandatory. Additionally, pharmacovigilance practices for these natural products are crucial to understanding the benefit, limitations, dosage regimen, and potential effects and how these modalities need to be modified during some periods.

Tables 1 and 2 summarize the main effects in the consulted clinical studies. Clinical studies were gathered accordingly to disease phase and main action. The ratio of trials* versus* nontrials included all the interventional clinical trials* versus* total of clinical studies.

Based on clinical study data, the following supplements can be used in the different phases of breast cancer history:

*(i) Preventive Effects*

  Phytotherapeutic products: flaxseed; green tea.
  Nutritional supplements: omega 3 polyunsaturated fatty acids (minimum daily dose of 2.5 g in women at high breast cancer risk).

*(ii) During Conventional Treatments after Diagnosis*

  Phytotherapeutic products:* U. tomentosa *as an adjuvant treatment of FAC (Fluorouracil, Doxorubicin, and Cyclophosphamide) regimen in Invasive Ductal Carcinoma Stage II;* Curcuma longa* as coadjuvant in docetaxel therapy; green tea as adjuvant of radiotherapy;* Viscum album* in side effects, recurrence, and antitumour activity.
  Nutritional supplements: vitamin C 500 mg + vitamin E 400 mg in side effects chemotherapy, vitamin D (necessary doses to target 30–50 ng/ml serum levels) + calcium, and vitamin D as adjuvant of aromatase inhibitor therapy; DHA as adjuvant in chemotherapy and side effects.

*(iii) Posttreatment (i.e., Survival Period)*

  Phytotherapeutic products:* Salvia *sp. associated with* Coriolus versicolor* improves immunologic function.

Based on the current literature we concluded that well-designed clinical studies are needed to obtain high level of evidence to accomplish recommendation guidelines.

## Figures and Tables

**Table 1:** The main clinical effects of the most common phytotherapeutic products used in breast cancer.

Nutritional supplements	Disease phases	Trials versus nontrial	Main effects from clinical studies	Type of clinical study	Ref.
*Echinacea *spp.	Risk	0/1	(i) Consumption not associated with increased risk for breast cancer	Prospective cohort	[30]
	Treatment	0/1	(i) Consumption not induced pharmacokinetic parameters alterations of docetaxel	Case control	[40]
	Prognostic	0/2	(i) Consumption associated with breast cancer survivors	Prospective cohort	[33, 34]

*Tabebuia impetiginosa*		0/0			

*Salvia *spp.	Prognostic	1/1	(i) Administration of *Salvia *extract (20 mg/Kg) and *Coriolus versicolor *(polysaccharopeptide 50 mg/Kg) promoted the immune function in posttreatment of breast cancer patients	Nonrandom clinical trial	[54]
	Side effects	1/1	(i) Administration of *Salvia *extract (IV) reduced skin ischemia and necrosis after mastectomy with less side effects when compared to anisodamine drug	Random clinical trial	[55]

*Uncaria *spp.	Side effects	1/1	(i) Uncaria extract (300 mg/day) reduced the neutropenia caused by chemotherapy and restored cell DNA damage in patients with Invasive Ductal Carcinoma Stage II	Random clinical trial	[56]

*Allium sativum *L.	Risk	0/1	(i) High consumption of *Allium* may reduce the risk of breast cancer	Case control	[57]
	Treatment	1/1	(i) Consumption does not affect the distribution of docetaxel. However, it can decrease docetaxel clearance in patients carrying a *CYP3A5∗1A* allele	Nonrandom pilot clinical trial	[58]

*Linum usitatissimum*	Risk	0/1	(i) Dietary consumption associated with a reduction in breast cancer risk	Case control	[59]
	Treatment	3/3	(i) Dietary consumption had the potential to reduce tumour growth in patients with breast cancer	Random double-blind placebo-controlled clinical trial	[60]
			(ii) Dietary consumption of flaxseed (25 g) to healthy volunteers during one menstrual cycle does not affect the angiogenin and VEGF levels in normal breast tissue but increase the endostatin levels similar to tamoxifen	Random clinical trial	[61]
			(iii) Flaxseed consumption does not affect the aromatase inhibitors activity	Random pilot clinical trial	[62]
	Prognostic	0/1	(i) High consumption of enterolignans (sunflower, pumpkinseeds, sesame, and flaxseeds origin) may have a better impact in postmenopausal breast cancer patient survival	Cohort	[63]

*Curcuma longa*		1/1	(i) Dose of 6 g/day of curcumin in combination with docetaxel (standard dose) demonstrated safe profile has a superior antitumour activity compared to docetaxel monotherapy	Nonrandom open-label clinical trial phase I	[64]

*Camellia sinensis*	Risk	1/6	(i) High tea consumption had no significant effect on the risk of several cancers, including breast cancer	Meta-analysis of prospective observational studies	[65]
			(ii) Green tea consumption was not associated with breast cancer risk in Japanese women	Cohort	[66]
			(iii) Regular green tea consumption can protect against breast cancer	Case control	[67]
			(iv) No association between plasma tea polyphenols and the risk of breast cancer in Japanese women	Nested case control	[68]
			(v) High epicatechin may be related to a reduced risk of breast cancer	Nested case control	[69]
			(vi) ECGC can prevent breast cancer by influencing the growth factor signalling, angiogenesis, and lipid metabolism mechanisms	Random placebo-controlled clinical trial phase IB	[70]
	Polymorphism	0/3	(i) Genetic polymorphism can influence polyphenols green tea metabolism and excretion	Nested case control	[71]
			(ii) Men carrying low-activity associated COMT genotype may retain more tea polyphenols	Cross sectional	[72]
			(iii) Green tea appeared to reduce breast cancer risk in Asian-American women with low-activity COMT alleles	Cross sectional	[73]
	Treatment	3/3	(i) EGCG potentiated efficacy of radiotherapy in breast cancer patients	Random pilot clinical trial	[74]
			(ii) Daily consumption of green tea (843 mg EGCG) is well tolerated by Caucasian postmenopausal women	Random double-blind placebo-controlled clinical trial	[75]
			(iii) Green tea extract phytosomes increased EGCG bioavailability and decreased tumour circulating biomarker revealing antiproliferative effects on breast cancer tissue	Nonrandom pilot clinical trial	[76]
	Prognostic	0/1	(i) Consumption of 5 or more cups of green tea a day may prevent breast cancer recurrence in early-stage (I and II) cancers	Meta-analysis of observational studies	[77]

*Ginseng*	Treatment	1/1	(i) Chinese herb formula including ginseng showed immunological improvement in breast cancer patients	Random clinical trial	[78]
	Prognostic	0/2	(i) Consumption of ginseng among breast cancer survivors was not associated with quality of life improvement	Cohort	[79]
			(ii) Regular consumption of ginseng 1.3 g/day may improve both overall and disease-free survival and enhance the quality life of Chinese women breast cancer survivors	Cohort	[80]

*Cimicifuga racemosa*	Risk	0/1	(i) No relationship was found between black cohosh consumption and increased risk of breast cancer	Meta-analysis of interventional and observational studies	[81]
	Prognostic	0/1	(i) Consumption can reduce risk of recurrence in patients taking tamoxifen	Retrospective cohort study	[82]

*Viscum album*	Treatment	1/1	(i) Mistletoe extract was highly effective in the tumour regression of breast cancer	Nonrandom pilot clinical trial	[83]
	Side effects	2/5	(i) Mistletoe therapy associated with CAF (Cyclophosphamide, Doxorubicin, and 5-Fluorouracil) chemotherapy resulted in clinical improvements of life quality in breast cancer patients	Random open-label pilot clinical trial	[84]
			(ii) *Viscum album* therapy during chemotherapy in the early-stage breast cancer patients increased the life quality, may prevent neutropenia, and did not influence the frequency of relapse or metastasis within 5 years	Prospective noninterventional follow-up study of a clinical trial	[85]
			(iii) Standardized aqueous mistletoe extracts therapy was well tolerated and reduced the side effects of chemotherapy, resulting in a significant stabilization of Health Related Quality of Life	Prospective cohort	[86]
			(iv) Mistletoe intravenous administration (1 and 5 mg) during chemotherapy had no significant effect on granulocyte function but reduced chemotherapy-related side effects	Random clinical trial phase II	[87]
			(v) Standardized mistletoe extract therapy improved quality of life and significantly reduced side effects of the disease/treatment	Prospective cohort	[88]

COMT: catechol-O-methyltransferase; VEGF: vascular endothelial growth factor.

**Table 2:** The main clinical effects of the most common nutritional supplements used in breast cancer.

Nutritional supplements	Disease phases	Trials versus nontrials	Main effects from clinical studies	Type of clinical study	Ref.
Multivitamins and antioxidants	Risk	0/7	(i) Supplementation of multivitamins and antioxidants in postmenopausal women may protect women from developing breast cancer	Case control	[104]
			(ii) High frequency and long duration multivitamins consumption was associated with an increase of breast cancer risk	Prospective cohort	[105]
			(iii) Multivitamins consumption was not associated with breast cancer risk	Case control	[106]
			(iv) Little inverse association between the use of multivitamins among white women and no evidence of reduced breast cancer risk among black women were reported	Case control	[107]
			(v) No association was verified between dietary intake of antioxidant vitamins and breast cancer risk	Case control	[108]
			(vi) Dietary intake of beta-carotene, vitamin C, and vitamin E was not related to breast cancer risk in pre- nor postmenopausal women	Prospective cohort	[109]
			(vii) Dietary antioxidant was associated with a lower risk of breast cancer and reduced mortality rate	Prospective cohort	[110]
	Prognostic	0/4	(i) Use of multivitamins by postmenopausal women with invasive breast cancer had lower breast cancer mortality than nonusers	Prospective cohort	[111]
			(ii) Posttreatment use of antioxidant supplements was associated with an improved survival in breast cancer patients from the United States and China	Meta-analysis of cohort studies	[112]
			(iii) Consumption of multivitamins improved outcomes related to breast cancer recurrence and survival after two years after diagnosis	Cohort	[113]
			(iv) Breast cancer survival was not improved by multivitamin treatment in nonmetastatic breast cancer diagnosed women	Cohort	[114]

Vitamin A and carotenoids	Risk	0/7	(i) No significant association was established between plasma retinol and vitamin A and breast cancer risk	Meta-analysis of case-control studies	[115]
			(ii) Plasma *β*-carotene was inverse associated with overall cancer risk, including breast cancer	Nested case control	[116]
			(iii) High carotenoids consumption may reduce breast cancer risk in premenopausal but not in postmenopausal	Case control	[117]
			(iv) Dietary intake of lycopene, beta-carotene, and beta-cryptoxanthin was associated with a lower breast cancer risk among Chinese women. No association was found for alpha-carotene and lutein/zeaxanthin	Case control	[118]
			(v) Serum alpha-carotene and beta-carotene were inversely associated with breast cancer risk	Prospective cohort	[119]
			(vi) Dietary intake of alpha-carotene, beta-carotene, and lycopene are inversely associated with invasive breast cancers risk. No association was observed with the intake of lutein + zeaxanthin and beta-cryptoxanthin	Prospective cohort	[120]
			(vii) Higher concentrations of plasma *β*-carotene and *α*-carotene were associated with a lower breast cancer risk	Nested case control	[121]
	Prognostic	0/1	(i) Positive relationship was reported between a high plasma carotenoids levels and breast cancer survivals	Cohort	[122]

Vitamin C	Risk	0/3	(i) High dose vitamin C intake (>1000 mg) was associated with a history of breast cancer	Cross sectional	[123]
			(ii) High dietary vitamin C intake was associated with an increased breast cancer risk among postmenopausal women	Cross sectional	[124]
			(iii) Plasma vitamin C was inversely associated with breast cancer risk	Meta-analysis of observational studies	[115]
	Prognostic	0/2	(i) Prediagnosis intake was positively associated with breast cancer survival while postdiagnosis was not	Cohort	[125]
			(ii) Postdiagnosis vitamin C supplement or dietary intake was associated with a reduced risk of breast cancer-specific mortality	Meta-analysis of cohort studies	[126]
	Side effects	1/4	(i) Supplementation of vitamin C (500 mg) and vitamin E (400 mg) during tamoxifen treatment reduced the tamoxifen-induced hypertriglyceridemia	Cohort	[127]
			(ii) Supplementation of vitamin C (500 mg) and vitamin E (400 mg) restored antioxidant enzyme status and DNA damage lowered in breast cancer and chemotherapy	Random clinical trial	[128]
			(iii) The IV administration of 50 g twice a week decreased fatigue and insomnia and increased cognitive function in a woman with recurrent breast cancer undergoing weekly chemotherapy	Case report	[129]
			(iv) The IV administration of 7.5 g resulted in a significant reduction of complaints induced by the disease and chemo/radiotherapy, without side effects	Cohort	[130]

Vitamin E	Prognostic	0/1	(i) Vitamin E appears to be a factor in poor prognosis for breast cancer survival	Cohort	[131]
	Side effects	2/5	(i) Supplementation of vitamin C (500 mg) and vitamin E (400 mg) during tamoxifen treatment reduced the tamoxifen-induced hypertriglyceridemia	Cohort	[127]
			(ii) Supplementation of vitamin C (500 mg) and vitamin E (400 mg) restored antioxidant enzyme status and DNA damage lowered in breast cancer and chemotherapy	Random clinical trial	[128]
			(iii) Alpha-tocopherol acetate (400 mg) supplementation increased biomarkers of estrogen-stimulation when coadministrated with tamoxifen	Case control	[132]
			(iv) Association of 400 mg pentoxifylline and 100 mg of vitamin E after radiotherapy in breast cancer women may be used to prevent radiation-induced side effects	Random placebo-controlled clinical trial	[133, 134]

Vitamin D and calcium	Risk	2/41	(i) Vitamin D deficiency is highly prevalent in breast cancer patients	Cross-sectional analytical study	[135]
			(ii) No association was observed between vitamin D supplementation and breast cancer risk in postmenopausal women	Meta-analysis of random clinical trials	[136]
			(iii) No association was verified between vitamin D supplementation and breast cancer risk in young women	Case control	[137]
			(iv) No association was established between vitamin D intake and breast cancer	Cohort	[138]
			(v) Long-term calcium intake was not related to breast cancer risk	Prospective cohort	[139]
			(vi) Calcium intake from several sources was not associated with breast cancer risk in Chinese women	Case control	[140]
			(vii) No association was found between dietary intake of vitamin D and calcium and breast cancer risk	Cohort	[141]
				Case control	[142]
			(viii) No association was reported between daily use of 1000 mg of calcium carbonate and 400 IU of vitamin D3 and benign proliferative breast disease risk	Random placebo-controlled clinical trial	[143, 144]
			(ix) No association was verified between vitamin D3 serum levels and breast cancer	Nested case control	[145]
				Cohort	[146]
			(x) No association was established between vitamin D and calcium serum levels and breast cancer risk	Cohort	[147]
			(xi) Serum calcium levels was not related to breast cancer in Asian population	Cohort	[148]
			(xii) Dairy products were not associated with breast cancer risk	Case control	[149]
				Cohort	[150, 151]
			(xiii) UV light combined with dietary vitamin D intake was associated with a lower breast cancer risk in high latitudes	Cohort	[152]
			(xiv) Dietary vitamin D was associated with a decrease in breast cancer risk	Case control	[153]
			(xv) Vitamin D supplements demonstrated a protective effect in breast cancer risk compared with nonuser Pakistani women	Case control	[154]
			(xvi) Vitamin D intake protects from breast cancer risk in premenopausal women	Case control	[155]
			(xvii) Dietary vitamin D and calcium intakes were associated with a decrease in breast cancer risk	Case control	[156]
			(xviii) Dietary vitamin D and calcium intakes were inversely related to breast cancer risk	Meta-analysis of observational studies	[157]
			(xix) Breast cancer risk presented an inverse relationship between vitamin D intake in premenopausal and calcium intake in postmenopausal women	Case control	[158]
			(xx) Higher plasma vitamin D3 was associated with a decreased breast cancer risk for women with a lower BMI; in higher alcohol intakes, lower levels of vitamin D3 are associated with an increase in breast cancer risk	Nested case control	[159]
			(xxi) Serum vitamin D was associated with a decrease in breast cancer risk	Case control	[150, 160–166]
			(xxii) Daily intake of 600 mg calcium + 400 IU vitamin D and 30 ng/ml of serum vitamin D adequate to lower breast cancer risk	Dose-response meta-analysis of observational studies	[156]
			(xxiii) Higher plasma vitamin D and moderate physical activity are protective factor while family history and menopause are a risk factor	Case control	[167]
			(xxiv) Serum vitamin D levels > 27 ng/ml may reduce breast cancer risk in postmenopausal women but not in premenopause	Dose-response meta-analysis of prospective studies	[168]
			(xxv) Serum calcium were inversely associated with breast cancer in premenopausal women and the opposite occurred in overweight postmenopausal women	Prospective cohort	[169]
			(xxvi) Serum calcium and vitamin D3 levels were inversely associated with breast cancer risk	Meta-analysis of prospective studies	[170, 171]
			(xxvii) U-shape association between vitamin D plasma levels and cancer risk and inverse association with calcium serum levels were established	Cohort	[172]
			(xxviii) U-shape association was reported between vitamin D3 plasma levels and cancer risk and prognosis	Nested case control	[173]
	Polymorphism	0/5	(i) Presence of BB genotype of vitamin D receptor was associated with a significantly lower risk of advanced breast cancer	Case control	[174]
			(ii) GC and vitamin D receptor gene polymorphism relationship with breast cancer may be altered by menopausal status and type of cancer	Case control	[175]
			(iii) VDR polymorphism determines breast cancer risk	Case control	[176–178]
	Prognostic	0/8	(i) Vitamin D intake was not associated with breast cancer recurrence	Nested case control	[179]
			(ii) High calcium/magnesium ratio was related to an improved breast cancer survival	Cohort	[180]
			(iii) Breast cancer women with deficient vitamin D levels had an increased risk of recurrence and dead	Cohort	[181]
			(iv) Higher vitamin D serum levels may be associated with improved breast cancer survival but without statistical significance	Cohort	[182]
			(v) Lower serum vitamin D level was associated with aggressive subtypes of cancer	Case control	[177, 183, 184]
			(vi) Calcium serum levels was positively related to breast tumour aggressiveness	Prospective cohort	[185]
	Side effects	5/9	(i) Daily dose 400 UI vitamin D3 for 1 year during and after chemotherapy was not sufficient to increase vitamin D deficiency in breast cancer	Cohort	[186]
			(ii) No differences in aromatase inhibitors side effects were found between vitamin D3 daily doses of 600 UI and 4000 UI	Random double-blind clinical trial phase III	[187]
			(iii) Daily 10000 IU of vitamin D3 and 1000 mg of calcium supplementation in breast cancer patients with bone metastasis reduced elevated parathyroid hormone levels but had no beneficial palliative or bone resorption	Nonrandom clinical trial phase II	[188]
			(iv) Doses of 500–1500 mg calcium and 200–1000 IU vitamin D were insufficient to prevent bone loss	Systematic review of clinical trials	[189]
			(v) Vitamin D supplementation (50,000 IU/week) may reduce side effects of aromatase inhibitors	Cohort	[190]
			(vi) Weekly dose of vitamin D reduced aromatase inhibitor side effects	Random placebo-controlled clinical trial	[191]
			(vii) Vitamin D3 and calcium supplementation (2000 IU/1000 mg and 4000 IU/1000 mg) increased serum vitamin D3 concentrations and improved arthralgia induced by aromatase inhibitors	Nonrandom clinical trial	[192]
			(viii) Serum vitamin D3 target of 40 ng/ml reduced arthralgia related to aromatase inhibitors	Cohort	[193]
			(ix) Vitamin D supplementation may improve bone loss if target serum levels achieve 30 ng/ml	Cohort	[194]

B complex vitamins	Risk	1/31	(i) Superior plasma folate levels may be associated with an increased breast cancer risk in women with a *BRCA1/2* mutation	Prospective Cohort	[195]
			(ii) Daily supplementation of folic acid (2.5 mf of folate), vitamin B_6_ (50 mg), and vitamin B_12_ (1 mg) had no effect on overall risk of total invasive cancer or breast cancer among women during the folic acid fortification era	Random, double-blind, placebo-controlled trial	[196]
			(iii) Dietary folate intake has no significant effect on the breast cancer risk. Daily 220 *μ*g increment in dietary folate intake was not associated with the risk of breast cancer	Systematic review and meta-analysis of observational studies	[197]
			(iv) Dietary folate intake and blood folate levels did not associate with breast cancer risk and this did not vary by menopausal status or hormonal receptor status	Meta-analysis of prospective and case-control studies	[198]
			(v) Weak evidence of an inverse relationship between breast cancer risk and riboflavin intake and a positive association with vitamin B_12_ were established. No association varied by tumour hormone receptor status	Prospective cohort	[199]
			(vi) No evidence that high folate intakes (dietary and supplementation) before diagnosis adversely affect breast cancer survival after chemotherapy	Prospective cohort	[200]
			(vii) Scientific evidence does not support the hypothesis that higher dietary folate intakes reduce the risk for breast cancer	Systematic review of clinical studies	[201]
			(viii) Little or no association was reported between of plasma folate, pyridoxal 5-phosphate (i.e., the principal active form of vitamin B_6_), and vitamin B_12_ levels and breast cancer risk	Prospective cohort	[202]
			(ix) Unclear association between plasma folate and vitamin B_12_ levels and overall breast cancer risk	Prospective cohort	[203]
			(x) The red blood cell folate levels were not associated with breast cancer risk	Case control	[204]
			(xi) Little or no association was shown between dietary folate intake and breast cancer risk; in addition, a dose-response meta-analysis suggested a J-shaped relationship between folate intake and breast cancer risk	Dose-response meta-analysis of prospective studies	[205]
			(xii) Dietary folate intake was not associated with breast cancer risk but may be inversely associated with ER-positive /PR-negative tumours in Swedish patients	Case control	[206]
			(xiii) Weak association was reported between dietary vitamin B_2_ intake and reduced breast cancer risk	Systematic review and meta-analysis of epidemiologic studies	[207]
			(xiv) Dietary folate and vitamin B_6_ intakes were inversely associated with breast cancer risk by both ER and PR status in Chinese women	Case control	[208]
			(xv) High dietary vitamin B_6_ intake is associated with a lower risk of developing ER-negative breast cancer in Taiwanese women	Case control	[209]
			(xvi) High dietary folate intake was associated with a lower incidence of postmenopausal breast cancer	Prospective cohort	[210]
			(xvii) High dietary folate intake was associated with a reduced breast cancer risk in French women. Vitamin B_12_ intake may alter this association	Prospective cohort	[211]
			(xviii) Dietary folate intake was inversely associated with breast cancer risk. Dietary methionine, vitamin B_12_, and vitamin B_6_ (i.e., folate cofactors) intakes were not independently related to risk of breast cancer; however, they may modify the effect of folate	Case control	[212]
			(xix) Higher dietary folate intake is slightly associated with a lower risk for ER-negative breast cancer, and high vitamin B_12_ and methionine intakes are marginally associated with a lower risk of ER-positive breast cancer among Hispanic and non-Hispanic white women in the southwestern US	Multicentered, population-based case control	[213]
			(xx) High dietary folate intake may diminish breast cancer risk and this relationship may differ by menopausal and ER/PR status in Chinese patients	Prospective cohort	[214]
			(xxi) Adequate folate intake may reduce the increased breast cancer risk	Meta-analysis of prospective and case-control studies	[215]
			(xxii) Inverse association was verified between plasma folate levels and breast cancer risk was highly among women consuming at least 15 g/day of alcohol. Plasma vitamin B_12_ levels were inversely associated with breast cancer risk among premenopausal women but not among postmenopausal women. Plasma homocysteine levels was not associated with breast cancer risk	Nested case control	[216]
			(xxiii) Serum pyridoxal 5-phosphate (i.e., the principal active form of vitamin B_6_) levels and dietary methionine intakes are associated with a reduced breast cancer risk, especially in postmenopausal women	Dose-response meta-analysis	[217]
			(xxiv) High plasma vitamin B_6_ levels may diminish the breast cancer risk, particularly of ER-positive breast cancer; high plasma riboflavin levels may decrease the risk of breast cancer in premenopausal but not postmenopausal women; and plasma homocysteine and the other B vitamins (e.g., folate and vitamin B_12_) levels do not appear to influence breast cancer risk	Nested case control	[218]
	Polymorphism	0/7	(i) Association between MTHFR C667T polymorphism and breast cancer risk and no association between dietary folate intake and MTHFR C677T polymorphisms were established	Case control	[219]
			(ii) Neither dietary folate and related B vitamins intakes nor MTHFR or MTR genotypes were overall associated with breast cancer risk in Japanese women. Associations of nutrients with breast cancer risk did not differ by hormone receptors status	Case control	[220]
			(iii) Association was observed between MTHFR C677T and MTR A2756G polymorphisms and breast cancer risk. Dietary folate, vitamin B6, and vitamin B12 intakes influence these associations	Case control	[221]
			(iv) Significant association was observed between MTHFR C667T polymorphism, dietary folate, and vitamin B_6_ intake and breast cancer risk and an interaction between MTHFR C667T polymorphism and folate intake on the breast cancer risk	Case control	[222]
			(v) Vitamin B_12_ seems to reduce the risk of breast cancer, and MTHFR 665TT was associated with an increased breast cancer risk. Folate and vitamin B_12_ intakes and MTHFRC677T and MTHFR A1298C polymorphisms showed no association with breast cancer risk. THFR C665T genotype and low vitamin B6 intake are associated with an increased in breast cancer risk among Chinese population	Case control	[223]
			(vi) Neither dietary folate, vitamin B_6_, or vitamin B_12_ intakes nor MTHFR polymorphisms were independently associated with breast cancer risk. Increased breast cancer risk was observed in MTR 2756GG genotype and in premenopausal women with high folate intake among Brazilian women	Case control	[224]
			(vii) Dietary folate and cobalamin intakes are inversely associated with methylated retinoic acid receptor-beta (RARB) and breast cancer-1 (BRCA1) genes. High dietary riboflavin and pyridoxine intakes are associated with increased methylation in the RARB promoter in Iranian patients	Prospective cohort	[225]
	Prognostic		(i) Dietary vitamins B_1_ and B_3_ intake was associated with improved survival among women with breast cancer. MTHFR 677T polymorphism reduced all-cause mortality and breast cancer-specific mortality	Cohort	[226]
			(ii) Superior prophylactic effect of niacinamide compared to standard care for avoiding cutaneous symptoms and maintaining life quality of breast cancer patients while undergoing cytostatic treatment	Multicentre randomized crossover trial	[227]

Omega 3 PUFAs	Risk	4/16	(i) Comparable doses of marine *ω*-3 in dietary fish or in supplement provided increased plasma EPA and DHA in plasma, erythrocyte membranes, and breast adipose in women with a high risk of breast cancer. Increases in breast adipose EPA and DHA were the same for both groups	Random clinical trial	[228]
			(ii) Total PUFAs were associated with increased overall and breast cancer risk in the placebo group, whereas this relationship was not observed in the antioxidant-supplemented group (antioxidants preserve essential PUFAs from peroxidation)	Nested case control	[229]
			(iii) No association was observed between EPA and DHA intake from fish oil supplements and breast cancer outcomes. Marine fatty acids from food reduced risk of additional breast cancer events and all-cause mortality in breast cancer survivors	Cohort	[230]
			(iv) No association was established between dietary total fat and fatty acids, including *ω*-3 PUFAs and breast cancer risk	Meta-analysis of prospective cohort studies	[231]
			(v) No association was reported between total or individual marine *n*-3 PUFA in adipose tissue and breast cancer risk	Case cohort	[232]
			(vi) No association was observed between fish consumption and breast cancer risk	Meta-analysis of observational studies	[233]
				Prospective cohort	[234]
			(vii) Current use of fish oil may be inversely associated with ductal breast cancer risk in postmenopausal women	Cohort	[235]
			(viii) Fish oil consumption had a protective effect in breast cancer	Case control	[236]
			(ix) Omega 3 PUFAs presented a preventive action in postmenopausal women	Case control	[236]
				Cohort	[237]
			(x) Inverse relationship was established between dietary marine *n*-3 PUFA and breast cancer risk	Meta-analysis and systematic review of prospective cohort studies	[238]
			(xi) Higher omega 3 : omega 6 ratio intake and breast cancer risk had an inverse association	Meta-analysis of prospective studies	[239]
				Prospective cohort	[240]
			(xii) Consumption of high levels of *ω*-3 and low levels of *ω*-6 had a reduced breast cancer risk, compared to women who consume low levels of *ω*-3 and high levels of *ω*-6 among Long Island, New York, residents	Case control	[241]
			(xiii) A minimum daily dose of 2.52 g EPA + DHA is required to increase their concentrations in breast adipose tissue. Daily doses up to 7.56 g of DHA and EPA were well tolerated with optimal compliance. BMI and baseline fatty acid concentrations modulated the dose-response outcomes of *ω*-3 PUFAs supplements on serum EPA and DHA and breast adipose tissue DHA in women at high risk of breast cancer	Random open-label dose-finding study	[242]
			(xiv) Primary prevention trial of high dose EPA and DHA ethyl esters at a daily dose of 3.36 g (1860 mg EPA +1500 mg DHA) resulted in a good uptake, excellent tolerability, and retention in postmenopausal women. Increase *ω*-3 PUFAs (EPA+DHA): *ω*-6 AA ratio in erythrocyte and benign breast tissue phospholipids provided a favourable modulation in several biomarkers of breast cancer risk and inflammatory process	Phase II pilot study	[243]
			(xv) Increase in plasma DHA was associated with a decrease in absolute breast density (i.e., a validated biomarker of breast cancer risk) but only in obese women (BMI > 29)	Open-label random clinical trial	[244]
	Treatment	3/3	(i) Combination of omega 3 (4 g) and raloxifene (30 mg) reduced IGF-1 levels and improved serum lipids, antioxidant, and anti-inflammatory activities	Random controlled placebo clinical trial	[245, 246]
			(ii) Combination of DHA to an ROS-generating chemotherapy regime was safe and retained significant antitumour activity in metastatic breast cancer patients with high plasma DHA incorporation	Pilot open-label single-arm phase II clinical trial	[247]
			(iii) High dose EPA and DHA supplementation (4 g/day) for 3 months increased serum EPA and DHA levels and total and long-chain *ω*-3 PUFAs and decreased arachidonic acid, total and long-chain *ω*-6 PUFAs, and the *ω*-6 : *ω*-3 PUFAs ratio compared to placebo. This dose also reduced bone resorption	Random, double-blind, placebo-controlled pilot study	[248]

AA: arachidonic acid; BMI: body mass index; BRCA1: breast cancer-1 gene; DHA: docosahexaenoic acid; EPA: eicosapentaenoic acid; ER: oestrogen receptor; GC: gene encoding vitamin D binding protein; IGF-1: insulin-like growth factor; IV: intravenous; MTHFR: 5,10-methylenetetrahydrofolate reductase; MTR: methionine synthase; PR: progesterone receptor; PUFAs: polyunsaturated fatty acids; RARB: retinoic acid receptor-beta gene; VDR: vitamin D receptor.
